# Engrams Formed in Virtual Reality Exhibit Reduced Familiarity Upon Retrieval: Electrophysiological Correlates of Source Memory Retrieval Indicate Modality‐Dependent Differences in Recognition Memory

**DOI:** 10.1111/ejn.70239

**Published:** 2025-09-07

**Authors:** Joanna Kisker, Marius Soethe, Jonas Sieverding, Leon Lange, Merle Sagehorn, Benjamin Schöne, Thomas Gruber

**Affiliations:** ^1^ Experimental Psychology I, Institute of Psychology Osnabrück University Osnabrück Germany; ^2^ Swartz Center for Computational Neuroscience, Institute for Neural Computation University of California San Diego San Diego USA; ^3^ Department of Psychology Norwegian University of Science and Technology Trondheim Norway

**Keywords:** dual‐process theory, event‐related potentials, phase‐amplitude coupling, source memory, virtual reality

## Abstract

Initial findings linking Virtual Reality (VR)‐based encoding to increased recollection at retrieval remain inconclusive due to heterogeneous study designs and dependence on behavioral data. To clarify under which circumstances VR‐based encoding affects or enhances episodic memory retrieval, the fundamental question remains whether the encoding modality, i.e., VR or 2D‐desktops (PC), functions as a source for recollection, independent of further contextual factors. Specifically, the electrophysiological correlates (EEG) of item and source memory could objectively determine whether source retrieval fosters recollection and attenuates familiarity of VR‐encoded information (i.e., VR‐engrams) compared to PC‐encoded information (i.e., PC‐engrams).

To this end, participants incidentally encoded everyday objects in VR and on a 2D desktop in a within‐subjects design, followed by unannounced old/new and source identification tasks. Our results indicate that encoding modality affects item memory only to a limited degree: Recognition memory performance, alongside the electrophysiological markers of item memory, i.e., the frontal and parietal old/new effects (FN400, LPC) and the theta band response, yielded comparable results for both engrams. Yet source memory differs depending on the encoding modality: The late posterior negativity indicated a shift towards recollection regarding the retrieval of VR‐engrams compared to PC‐engrams. This shift might result from attenuated familiarity with VR‐engrams, particularly reflected in the alpha band and phase‐amplitude coupling of theta and gamma band. In conclusion, encoding modality functions to some degree as a relevant source for recognition memory. Yet our results propose that familiarity is more strongly affected than recollection if contextual information beyond dimensionality is matched between encoding modalities.

## Graphical abstract

The study examines whether the retrieval of virtual reality (VR)‐engrams is more profoundly based on recollection than on familiarity compared to PC‐engrams, and whether the encoding modality functions as a relevant source information for episodic memory retrieval. To this end, participants incidentally encoded everyday objects in VR and on a conventional PC in a within‐subjects design. In a subsequent unannounced recognition task, participants discriminated between old objects presented during encoding and new objects, functioning as distractors (old/new task; item memory). If a stimulus was rated old, participants were asked to indicate the object's source by means of the encoding modality, i.e., VR or PC (source identification task; source memory). Our results indicate that encoding modality affects item memory only to a limited degree, whereas source memory differs depending on the encoding modality. Our results propose that familiarity is more strongly affected than recollection if contextual information beyond dimensionality is matched between encoding modalities.

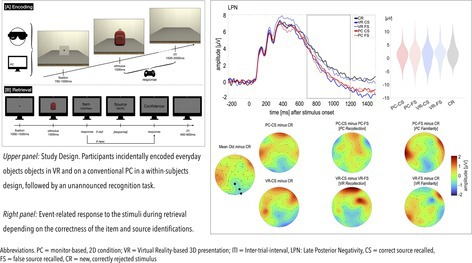

**Abbreviations**
ABR: (induced) alpha band response
CR: correct rejection
CS: correct source retrieved
EEG: electroencephalography
EOG: electrooculogram
FS: false source retrieved
GBR: (induced) gamma band response
HMD: head‐mounted display
MI: modulation index
PAC: phase‐amplitude coupling
PC: personal computer
TBR: (induced) theta band response
VR: virtual reality

## 1 Introduction

Virtual Reality (VR) is proposed to possess certain features that are considered decisive for episodic memory (Serino and Repetto 2018), e.g., by providing a sensory‐rich and meaningful context (Park and Donaldson 2019; Slater and Wilbur 1997). Yet empirical findings remain inconclusive regarding VR's effect on retrieval performance and on the underlying processes. An overarching trend proposes that engrams, i.e., the pattern of widespread neural activity representing encoded information, formed under VR‐based conditions (hereafter: VR‐engrams) result in enhanced memory retrieval compared to engrams formed under desktop‐based conditions (hereafter: PC‐engrams; Serino and Repetto 2018; Smith 2019; Smith and Mulligan 2021). For example, superior retrieval performance for VR‐engrams compared to PC‐engrams after incidental encoding was found regarding the retrieval of photorealistic video scenes (Schöne et al. 2019, 2021), tasks described and performed within VR (Harman et al. 2017), as well as 2D stimuli presented within VR (Krokos et al. 2019). Yet further studies break this trend, i.e., report equal memory performance under both conditions (Cadet and Chainay 2020; Ernstsen et al. 2019; Kisker et al. 2021b). Given the relative novelty of using VR to examine episodic memory performance and processes, the aforementioned studies are heterogeneous not only in their results but also in their design, resisting a straightforward identification of the circumstances under which episodic memory retrieval differs or benefits from VR‐based encoding.

Possible reasons that contribute to this heterogeneity might be that (I) most studies do not differentiate the subprocesses leading to successful memory retrieval (see e.g., Cadet and Chainay 2020; Ernstsen et al. 2019; Schöne et al. 2019), (II) the results are predominantly based on behavioral data (see e.g., Harman et al. 2017; Kisker et al. 2021a; Schöne et al. 2021), and (III) the high variance of stimulus complexity across previous studies (see e.g., Kisker et al. 2021b; Krokos et al. 2019). Consequently, the present study aims to overcome some of these limitations by (I) accounting for the two complementary subprocesses constituting successful retrieval according to the framework of the Dual Process Theory of recognition memory, i.e., familiarity and recollection (Tulving 1985; Yonelinas 2002), (II) fostering the objectification of the behavioral results by analyzing the electrophysiological correlates of recognition memory, and (III) matching conditions beyond dimensionality, i.e., only varying two‐ and three‐dimensionality between conditions to provide a fundamental examination of effects depending on the encoding modality per se. Visual quality and context, the way of presentation, and the extent of possible interactions were matched, respectively. In the following, we will briefly review the related literature.

Firstly, initial studies propose that the *processes* underlying the retrieval of engrams formed under either modality differ rather than the behavioral outcomes reflecting memory *performance* (Kisker et al. 2021a, 2021b; Schöne et al. 2019). In particular, the retrieval of VR‐engrams might more strongly rely on recollection, whereas the retrieval of PC‐engrams was proposed to rely more strongly on familiarity (Kisker et al. 2021a, 2021b). These complementary processes are postulated in the framework of the Dual‐Process Theory of recognition memory (Tulving 1985). Familiarity is usually defined as recognition based on an indistinct feeling of oldness, lacking the retrieval of specific details about the circumstances under which the memory was obtained (Kwon et al. 2023; Rugg and Curran 2007; Yonelinas 2002). In contrast, recollection is associated with the retrieval of contextual information on the encoding context and conditions under which the engram was formed (Kwon et al. 2023; Rugg and Curran 2007; Yonelinas 2002). While familiarity occurs relatively automatically, recollection is a more controlled process in comparison (Yonelinas 2002). Both processes are commonly examined in old/new or remember/know paradigms related to item memory, asking participants to differentiate between items encoded in a preceding study phase and new, unknown items. Remember/know paradigms further disentangle whether the item was recognized by familiarity (know) or vividly recollected by recalling additional contextual information (remember). However, the judgments on the retrieval processes rely on the participants subjective awareness of, and access to these processes (Yonelinas 2002), neglecting to control for the accuracy of this awareness (Migo et al. 2012). To overcome the latter, old/new tasks can be complemented with source identification tasks. These tasks objectively dissociate recollection and familiarity by testing associative information regarding contextual or encoding details, i.e., source memory (Jacoby 1991; Kwon et al. 2023; Wynn et al. 2024). Source memory can be characterized by various elements related to the encoding context, e.g., spatial–temporal information (Bröder and Meiser 2007), or the encoding modality (Johnson et al. 1993a). If remembering a stimulus includes correct recall of source information, this is considered a hallmark of recollection. Vice versa, remembering a stimulus but no or false source information indicates familiarity (Migo et al. 2012).

Most fundamentally for memory studies relying on the VR approach, the question arises whether the presentation medium used for encoding, i.e., VR or 2D desktops, respectively, functions as a relevant source for retrieval and particularly for recollection, i.e., whether information about the presentation modality is bound to the respective stimulus during encoding and available during retrieval. This also raises the question whether source retrieval might lead to altered or even better memory performance regarding VR‐engrams. In other words, if VR environments per se offer richer information during encoding, it is necessary to determine the effect of the presentation modality on the retrieval processes as a first step. Only this baseline will allow for a subsequent differentiation of the extent to which information beyond modality characterizing the encoding episode affects retrieval. Initial behavioral studies report a tendency for stimuli encoded in VR to be misattributed to reality more often than those encoded on screens (Bonnail et al. 2024; Rubo et al. 2021). These results indicate that the encoding context generally functions as a relevant source and differs depending on modality, with VR stimuli being processed relatively similar to real‐life stimuli (Rubo et al. 2021). However, like the majority of comparisons of the retrieval of VR‐ and PC‐engrams, these studies are based on subjective, behavioral data and leave the question about the underlying processes unresolved.

Consequently, the current study builds on analyzing the neural correlates of recognition memory to foster the objectification of the behavioral results. Examining item memory in the human EEG, familiarity has been associated with a frontal old/new effect in the event‐related potential (ERP) occurring ~ 300–500 ms after stimulus onset, also known as the FN400 (Kwon et al. 2023; Mecklinger 2000; Rugg and Curran 2007). In contrast, recollection is more strongly associated with a parietal old/new effect occurring ~ 500–800 ms after stimulus onset, also labeled the late positive component (LPC; Johansson and Mecklinger 2003; Kwon et al. 2023; Rugg and Curran 2007). Whereas the FN400 is less negative going for old compared to new stimuli, the parietal LPC is more positive going regarding old stimuli (Rugg and Curran 2007). Complementing these effects, the induced, i.e., non‐phase‐locked oscillatory activity offers further insights into the neural mechanisms underlying mnemonic processing which are not precisely time‐locked to stimulus onset (Graetz et al. 2019; Gruber et al. 2008; for review see e.g., Köster and Gruber 2022). In particular, the induced theta band response (TBR, ~ 3–7 Hz) is related to both encoding and retrieval processes (Hsieh and Ranganath 2014). For example, increased pre‐stimulus TBR predicted subsequent successful recall (Addante et al. 2011; Guderian et al. 2009), and post‐stimulus TBR is indicative of the accurate recollection of the encoding context (Addante et al. 2011). Moreover, enhancing the TBR by entrainment prior to memory tests increased source memory performance (Roberts et al. 2018). Albeit the TBR's broad implications for mnemonic processes (for review see e.g., Hsieh and Ranganath 2014), most relevant for the study at hand is its association with effortful and explicit control during cognitive processes (Graetz et al. 2019; Gruber et al. 2008; Guderian and Düzel 2005; Klimesch, Doppelmayr, Pachinger et al. 1997; Klimesch, Doppelmayr, Schimke et al. 1997). In old/new paradigms, the TBR yields a higher response to old stimuli compared to new stimuli, mirroring effortful cognitive control needed to retrieve engrams (Friese et al. 2013; Guderian and Düzel 2005; Hsieh and Ranganath 2014; Klimesch 1999; Nyhus and Curran 2010). While this effect has primarily been linked to successful retrieval, it is also related to the recollection of personal events (Graetz et al. 2019; Gruber et al. 2008; Guderian and Düzel 2005). As a complement, the induced alpha band response (ABR, ~ 8–12 Hz) desynchronizes in response to mental activity (Clayton et al. 2018; Klimesch, Doppelmayr, Schimke, et al. 1997) and is proposed to mirror memory load and attentional processes (Klimesch 1999; Sauseng et al. 2009). With respect to these processes, an initial examination of the electrophysiological correlates of item memory after VR‐based encoding replicated the canonical frontal difference in the TBR to old and new stimuli during retrieval of PC‐engrams, yet the retrieval of VR‐engrams was not accompanied by this effect. This led to the assumption that the TBR did not exclusively reflect retrieval *success* but retrieval *effort* as well (Kisker et al. 2021b). Yet the judgments on the retrieval processes, i.e., familiarity and recollection, relied on the participants subjective awareness of, and access to these processes (Yonelinas 2002), only allowing for indirect conclusions.

As outlined above, the relative contributions of familiarity and recollection to the retrieval of VR‐ and PC‐engrams can be evaluated even more objectively using source memory assessments. Processes associated with the retrieval of information that specify the retrieval context are reflected in the late posterior negativity (LPN). This deflection of the ERP is characterized by a more negative going response to old stimuli compared to new ones starting around 700–800 ms after stimulus onset (Cycowicz et al. 2001; Herron 2007; Johansson and Mecklinger 2003). Whereas the response‐locked LPN reflects action monitoring (Herron 2007; Mecklinger et al. 2016), the stimulus‐locked LPN is associated with the search for episodic information during an early stage (600–1200 ms) and with the maintenance of the retrieved episode at later stages (1200–1900 ms, Herron 2007). Albeit it is frequently, though not exclusively, reported that the LPN is not sensitive to the correctness of source retrieval (Johansson and Mecklinger 2003), it is related to the amount of context information available for integration into an item‐context representation during retrieval (Johansson and Mecklinger 2003; Mecklinger et al. 2007, 2016). The LPN might thus provide insights into whether one of both encoding modalities results in a higher amount of available context‐specific information, the latter being decisive for recollection (see e.g., Migo et al. 2012).

Beyond the ERP‐level, the dynamic interplay, i.e., the coupling between the GBR and the TBR has been proposed to reflect efficient mnemonic processing, mirroring top‐down regulation on currently activated cortical object representations (Graetz et al. 2019; Lisman and Jensen 2013). In particular, the induced gamma band response (GBR, > 30 Hz) marks both the formation and activation of the cortical object representation including perceptual, semantic, and conceptual features of a stimulus (Gruber et al. 2008; Tallon‐Baudry and Bertrand 1999). Hence, the mere activation of the neural object representation during retrieval might result in familiarity with this stimulus independent of contextual information (Gruber and Müller 2002; Schacter 1990; Tallon‐Baudry and Bertrand 1999). Importantly, the GBR has been found to be higher for false source retrieval compared to correct source retrieval, mirroring uncertainty or incorrectness about the retrieval decision (Wynn et al. 2024). Consequently, the phase‐amplitude coupling (PAC; Tort et al. 2010) between the GBR and TBR will be considered a measure of retrieval uncertainty or incorrectness. Based on Wynn et al. (2024), it is assumed that engrams with incorrect source identification are associated with the highest PAC. Since PAC has not yet been compared between modalities, we refrained from a directed hypothesis regarding modality‐dependent differences.

Last but not least, the heterogeneity of previous findings on VR's effect on memory retrieval might result from the broad range of stimulus complexity implemented across studies, limiting the comparability of effects found across these studies. Whereas the majority uses 3D objects (Cadet and Chainay 2020; Ernstsen et al. 2019; Kisker et al. 2021a) or 360° photos as stimuli (e.g., Ventura et al. 2019), stimulus complexity ranges from 2D pictures presented within VR (Krokos et al. 2019; Pastor and Bourdin‐Kreitz 2024) to detailed, photorealistic 3D‐360° videos (Schöne et al. 2019). Although these complex stimuli increase the relevance for, and transferability to real‐world processes, they preclude the isolation of factors underlying potential changes in memory processes. For instance, if photorealistic 3D‐360° scenes are better remembered than 2D scenes, it remains unclear whether this advantage arises from the three‐dimensionality per se or from the increased memorability of complex scenes in 3D, i.e., whether the effect is modality‐dependent, or rather depends on an interaction of content and modality. Consequently, a foundational understanding of whether the mere three‐dimensionality of the stimuli yields altered or even superior memory retrieval compared to 2D presentation on a desktop needs to be obtained. Only this reference point allows for a subsequent differentiation of how and why additional complex information from the encoding episode beyond modality affects recollection‐based retrieval.

In summary, our study examines whether the retrieval of VR‐engrams is objectively more profoundly based on recollection than on familiarity compared to PC‐engrams, and whether the encoding modality functions as a relevant source for episodic memory retrieval driving the aforementioned differences in retrieval processes. To this end, participants incidentally encoded everyday objects (e.g., toys, furniture, office items) using a VR headset (3D) and a conventional desktop (PC) in a within‐subjects design. In a subsequent unannounced recognition task, participants discriminated between old objects presented during encoding and new objects, functioning as distractors (old/new task; item memory). If a stimulus was rated old, participants were asked to indicate the object's source by means of the encoding modality, i.e., VR or PC (source identification task; source memory).

Since the TBR and the parietal LPC old/new effects are proposed to mirror recollection, they were hypothesized to be more pronounced for the retrieval of VR‐engrams compared to PC‐engrams. The FN400, related to familiarity, was proposed to be less pronounced during the retrieval of VR‐engrams compared to PC‐engrams. In case the encoding modality functions as a memory source, the LPN, reflecting the retrieval of source‐specifying attributes, was expected to be most pronounced for VR‐engrams recalled with correct source attribution. In the same train of thought, we expected different levels of PAC between TBR and GBR between VR‐engrams and PC‐engrams. Last but not least, we expected better retrieval performance of VR‐engrams compared to PC‐engrams despite the mixed literature background for this hypothesis, given that the three‐dimensional encoding context might provide richer contextual information fostering recollection during retrieval.

## 2 Methods

### 2.1 Sample Size and Participants

The study was approved by the local ethics committee of Osnabrück University (Ethik‐58/2023). All participants gave informed written consent and were blind to the research question. They received either partial course credits or 15€ for participation. A required sample size of 13 participants was determined using G*Power (Faul et al. 2007). The power analysis was based on the parameters for a 1 × 5 rmANOVA, and the effect size for the calculation was estimated from a previous study using a similar design but applying no VR condition (Gruber et al. 2008). The effect sizes *η* ^*2*^ from the analyses of both frequency bands of main interest, namely the TBR (*η* ^*2*^ *=* 0.341) and the GBR (*η* ^*2*^ *=* 0.303), were averaged (*η* ^*2*^ *=* 0.323) and converted to *f* = 0.69 via G*Power, which classifies as a large effect (Cohen 1988). Since the study used for the estimate is not a VR study, the effect size *f* was conservatively estimated as the lower bound of a large effect (*f* = 0.4). To compensate for potential technical problems and to establish comparability with other VR studies, we increased the sample size to 30 participants.

In total, 38 participants were recruited from Osnabrück University. An anamnesis was obtained from all participants by a short interview by the investigator, during which no participant reported suffering from psychological or neurological conditions. All participants had normal or corrected‐to‐normal sight. Four participants had to be excluded due to technical issues during the experiment. One participant canceled the experiment due to motion sickness; one participant was excluded due to missing data; and one participant had to be excluded due to unacceptable EEG data quality (blinks in 90% of the trials). After excluding these seven datasets, the final sample for analyses comprised 31 data sets (*M*_age_ = 22.61; *SD*_age_ = 3.90; 30 right‐handed, one without distinct handedness; gender: 23 female, 8 male; all cisgender). 25 participants had prior experience with VR head‐mounted displays but only one used them on a regular basis. 15 participants used computers regularly for entertainment, i.e., gaming.

### 2.2 Stimulus Material

**FIGURE 1:**
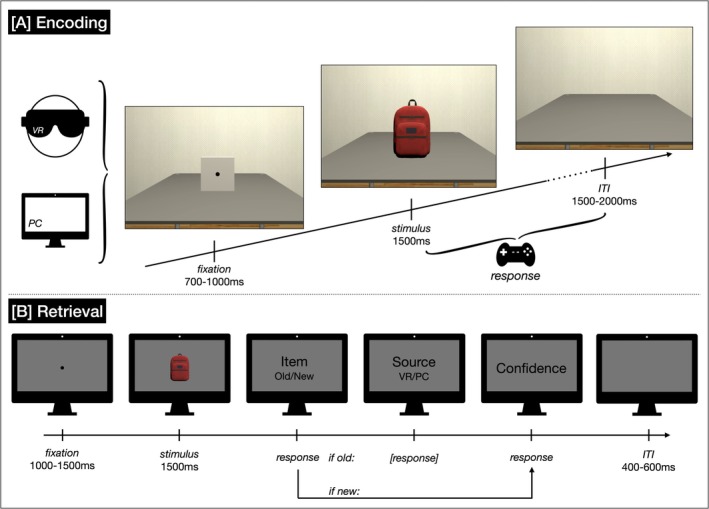
Schematic representation of the experimental procedure. *Note.* [A] Participants encoded 100 stimuli each in VR and on a PC in a within‐subjects design. The encoding phase took approximately 25 min in total, including three short breaks. [B] The retrieval task took approximately 40 min, including two breaks. Participants were asked to work on a Sudoku for 10 min between encoding and retrieval for inference of working memory by means of rehearsal.

The stimuli used were 305 3D objects obtained from three databases (Downs et al. 2022; Peeters 2018; Tromp et al. 2020). The reason for using objects from different databases was that none of the databases alone provided a large enough number of divergent stimuli. Five objects were used exclusively for instruction and training trials. They did not semantically overlap with the experimental trials. The training aimed to familiarize participants with the overall procedure of the experiment. Of the remaining 300 objects used, 50% depicted objects that would fit into a shoe box in their life‐size, while the other 50% would be larger than a shoe box in life‐size (*cf*. behavioral task & Figure 1).

### 2.3 Procedure

The experiment was conducted as a within‐subjects design and followed the conventional procedure of source memory paradigms, thus starting with an incidental encoding task, followed by an unannounced recognition memory task. For the encoding task, participants subsequently completed a VR‐ and a conventional PC‐condition in random order. Both conditions were created using Unity 5 (version 2023.2.1f1; Unity Technologies, San Francisco, USA) and the field of view of the virtual camera was set to the default of 60° in both conditions. For the VR‐condition, participants were equipped with the HTC Vive Pro 2 head‐mounted display, which offers a resolution of 2448 × 2448 pixels per eye and a frame rate of 90 Hz. Within the virtual environment, they were seated at a grey table with a distance of 90 cm to the position at which the stimuli would be presented. For the PC‐condition, participants were seated in front of a 55‐in. screen at a distance of 70 cm with a resolution of 1920 × 1080 pixels and a 60‐Hz frame rate. The same virtual environment, including the table, was depicted in 2D. The viewing angle of both conditions was matched via the size of the fixation cube, resulting in a viewing angle of 12°.

Each condition consisted of the five training trials and 100 experimental trials. For each subject, the 300 stimuli were randomly divided into subsets of 100 VR encoding trials, 100 PC encoding trials, and 100 distractors for retrieval, with each of these subsets containing 50 objects larger and 50 objects smaller than a shoebox regarding their real‐life size.

Participants were verbally instructed before completing the training trials and were given the opportunity to clarify any uncertainties about their task before the experimental trials were started. After 50 experimental trials, a two‐minute break was scheduled automatically to prevent fatigue and muscular tension. Each trial consisted of a fixation (700–1000 ms), the presentation of an object (1500 ms) and an interstimulus interval (1500–2000 ms; see Figure 1, Encoding). To maintain participants attention on the stimuli, they were instructed to indicate as quickly as possible for each object whether it was larger or smaller than a conventional shoe box in its real‐life size using the shoulder buttons of a conventional gamepad. The assignment of the two button presses for “larger” or “smaller” was counterbalanced across participants. In case participants had not yet responded after the object vanished, the trial was paused until the response was given before the next trial started. The start of the training trials, the start of the experimental trials, and the end of the break were indicated by a green cube showing a start symbol. Vice versa, the end of the training trials, the onset of the break, and the end of the experiment were indicated by a red cube showing a pause symbol. Each condition took about 12 min, including the break.

After participants completed both encoding conditions, there was a change of room to the EEG laboratory, where a 32‐channel electroencephalogram (EEG) and a four‐channel electrooculogram (EOG) were prepared. To avoid effects of potential rehearsal on the subsequent recognition task, participants were asked to complete a Sudoku on a tablet during the preparation of the EEG. Participants worked on the Sudoku either until they finished or until 10 min had passed (cutoff: earlier of both events).

EEG was recorded during the recognition memory task, during which participants were seated in an electrically shielded cabin (faraday cage). The instructions were given in writing on the screen and with the possibility to clarify any uncertainties before the experimental trials started. The unannounced recognition test comprised 2D pictures of the total set of the 300 3D objects. Objects presented during encoding in VR are denoted as “VR”, objects presented during encoding on the PC screen as “PC” and objects which were only presented during the retrieval task, and are thus unknown, are denoted as “CR” (correctly rejected items). All stimuli were presented on a conventional 24″ monitor (1020 × 1200 pixels resolution) on a grey background that matched the color of the table from the encoding session. The viewing angle was 7.96° horizontally and 3.49° vertically.

Each trial of the recognition task started with 1000–1500 ms fixation, followed by 1500 ms stimulus presentation. After stimulus offset, a set of rating scales was consecutively presented (see Figure 1, Retrieval). First, participants were asked to indicate whether they recognized the presented stimulus or not (old/new task). If participants categorized a stimulus as being old, they were subsequently asked to indicate whether they had previously seen it in VR or on the PC screen (source identification task). If they categorized the stimulus as being new, the source identification task was skipped. Last, participants were asked to indicate how confident they were about their answer (very unsure [1], unsure [2], sure [3], very sure [4]). The last rating scale was followed by an interstimulus interval of 600 ms (blank screen). The total recognition task took approximately 40 min. To prevent fatigue, participants were offered a pause after every 100 trials. They paused on average 37 s after 100 trials and 56 s after 200 trials (total pause on average 94 s). On completion of all trials, the EEG equipment was removed from the participants, and they were debriefed.

BOX 1 **Piloting of the delay phase between encoding and retrieval.**

**Rationale.** Previous studies comparing episodic memory performance and processes after VR‐based and PC‐based encoding vary in the delay phase between encoding and retrieval, particularly in whether the delay is long enough to include one night's sleep (see e.g., Kisker et al. 2021a, 2021b; Schöne et al. 2019). Therefore, the aim of our piloting was to test whether a delay long enough to include a sleep phase should be integrated in our main study.

**Methods.** Forty participants (*M*_*age*_ = 21.9, *SD*_*age*_ = 2.29; 31 females, 9 males) were randomly assigned to either one of four groups resulting from the cross‐combinations of the factors delay (*24 h delay, no delay*) and encoding modality (*VR, PC*), resulting in *n* = 10 per group. Participants were instructed to view 80 non‐semantic objects presented in 3D using an HTC Vive Pro 2 (*VR*) or in 2D on a standard desktop (*PC*), and to assign each object a fantasy name. Two randomly selected fantasy words were presented for each object to choose from. The incidental encoding task was followed by a 10 min distraction task. Afterwards, 50% of the participants were dismissed and asked to return after 24 h to complete the experiment (*24 h delay*), whereas the remaining 50% directly continued on (*no delay*). Participants were asked to perform an unannounced recognition memory task on a standard desktop. They were to discriminate between objects presented during encoding and new, unknown objects (old/new task). 160 trials were carried out with a 50:50 ratio of old and new objects. Additionally, participants were asked to indicate their confidence about each old/new decision (very unsure [1], unsure [2], sure [3], very sure [4]).

**Analyses and results**. To compare memory performance between groups, the proportion of correct answers and *d*‐prime, as well as the participants confidence, were compared between groups. A Bayesian rmANOVA with the factors delay (*24 h delay, no delay*) and encoding modality (*VR, PC*) was carried out per measure. Only descriptively, we observed higher values for both measures of memory performance regarding both *VR* groups compared to both *PC* groups, and slightly higher values for both *no delay* groups compared to both *24 h delay* groups. However, the Bayes Factor revealed moderate to anecdotal evidence for the H_0_, i.e., against group differences in the proportion of correct answers (*BF*_*10*_ = 0.36; *M* = 0.66, SD = 0.08), *d*‐prime (*BF*_*10*_ = 0.38; *M* = 0.59, SD = 0.33) and the participants confidence (*BF*_*10*_ = 0.30; *M* = 2.69, SD = 0.31).

**Conclusion**. Since the results did not imply group differences when varying the delay while keeping all other tasks and phases of the experimental procedure constant, we refrained from including a 24‐h delay in the main study but limited the delay to a distraction task.

### 2.4 Behavioral Data

The metrics from the encoding phase, i.e., response time and accuracy, are reported descriptively for completeness but not compared between conditions; the task's content was not directly linked to the recognition task. Performance and reaction time data were calculated across conditions, as well as separately for the VR‐condition and the PC‐condition.

To determine the participants memory performance depending on the encoding modality, the relative number of items correctly identified as old was calculated separately for stimuli encoded either in VR or on the PC. Congruently, the relative number of correct source identifications was calculated as the proportion of correct identifications in the number of performed ratings per encoding modality, as well as their confidence about these ratings. D‐prime (*d’*) was calculated as a sensitivity index, which quantifies how well old stimuli and distractors can be discriminated (*d’* = *z [hits] – z [false alarms];* Swets et al. 1961). In other words, *d’* estimates a participant's ability to discriminate between signal and noise independent of a response bias by considering both hits and false alarms. Yet, to control for potential response biases in the old/new task and the source task, respectively, criterion *C* was calculated as C = −0.5 * (z [hits] + z [false positive responses]) (Hautus et al. 2021; Macmillan 2002) per encoding modality and task. This measure indicates if participants tend to give a certain answer if uncertain. Negative values of *C* indicate liberal biases with a tendency to more hits and false alarm rates. Positive values indicate conservative biases with a tendency to more correct rejections and misses (Macmillan 2002).

Additionally, the participants confidence about their judgments was compared between the conditions of the source identification task (PC with correct source, PC‐CS; PC with false source, PC‐FS; VR with correct source, VR‐CS; VR with false source, VR‐FS; correct rejections, CR).

### 2.5 Electrophysiological Recordings and Preprocessing

An EEG with 32 active electrodes by Biosemi (Amsterdam, Netherlands) was recorded during the recognition task. Four further electrodes were applied for the assessment of an EOG. The electrodes were applied in accordance with the international 10–20 system. Additional CMS and DRL electrodes served as online reference and ground electrodes. To achieve a good signal‐to‐noise ratio, the live signal was checked for line noise and slow drifts and corrected before starting the retrieval task. The data was recorded at a sampling rate of 512 Hz. An online bandpass filter of 0.016–100 Hz was applied during the recording of the data. The EEG data was further preprocessed using MATLAB (version R2024a, MathWorks Inc) and EEGLAB (version 2024, Delorme and Makeig 2004).

#### 2.5.1 ERP

For the analyses of the ERP, the raw continuous data was filtered using the FIR band pass filter (eeglab function) with a 0.25‐Hz high‐pass filter and a 30‐Hz low‐pass filter. Each electrode was detrended separately before the data were segmented into epochs from 500 ms before stimulus onset to 1500 ms after stimulus onset. A baseline correction from −500 ms to stimulus onset was applied. No flatline channels were detected. Epochs including blinks were detected and removed using *statistical control of artefacts in dense array studies* (SCADS; Junghöfer et al. 2000; *M* = 10.16, SD = 12.38). An independent component analysis (ICA) was carried out, and the ICLabel function (Pion‐Tonachini et al. 2019) was used to identify and remove artefacts labeled as eye (> 0.85), muscle (> 0.9), or ECG (> 0.9). After removal of the respective components (*M* = 1.07, SD = 1.34), the data were re‐referenced to average reference. With respect to the old/new task, the trials were split into separate files containing trials in which PC objects were correctly identified as old (PC‐Old), VR objects correctly identified as old (VR‐Old), and new objects correctly rejected (CR), respectively, regarding the item memory (i.e., old/new) task. Depending on the participants responses, different numbers of trials were available per condition. To achieve equal signal‐to‐noise ratios across conditions, the condition with the fewest trials was identified per participant, and the larger categories were matched; i.e., random trials were removed until all conditions contained the same number of trials per participant. The time windows and regions of interest were derived from a current review, indicating an old/new effect in the time window from 300–500 ms after stimulus onset at frontal sensors (FN400) and a parietal old/new effect from 500–800 ms after stimulus onset at parietal sensors (LPC; Kwon et al. 2023; see also Mecklinger 2000; Rugg and Curran 2007). Typically, the FN400 is maximal around Fz and the LPC around Pz or POz. The regions of interest were thus chosen based on the aforementioned literature, i.e., based on the hypotheses. To identify the electrodes of relevance of our datasets, the conditions were averaged, and the maximum regional mean was determined, resulting in regional maxima at FC1 for the time window of the FN400 and the CP5 for the time window of the LPC. These electrodes were complemented by the surrounding electrodes, which deviated less than 0.5 SD from the regional maximum. However, the canonical electrodes Fz, Pz, and POz were not within this range and were thus not included. This procedure resulted in averaging across electrodes F3, FC1, FC5, C3, and Cz for analyses of the early time window and FC5, C3, and CP5 for analyses of the later time window.

With respect to the source identification task, the trials were split into separate files for VR objects with correct (VR‐CS) or false source retrieval (VR‐FS), PC objects with correct (PC‐CS) or false source retrieval (PC‐FS) and correct rejections of new objects (CR). As for the old/new task, the trial numbers were matched to the condition containing the fewest trials. Participants who had fewer than 15 trials per condition after matching were excluded from further analysis (*n* = 2). The time window and region of interest was derived from previous literature, i.e., based on hypotheses suggesting a source memory effect in the LPN from 700 to 1200 ms after stimulus onset and maximal around Pz and POz (Johansson and Mecklinger 2003; Mecklinger et al. 2007, 2016). Congruent with the FN400 and the LPC, the electrodes of relevance were adapted by identifying the regional minimum, which was O2. This regional minimum was complemented by electrodes within 0.5 SD, which included Pz and PO4 into analyses. POz was not available in our electrode setup.

#### 2.5.2 Spectral Power Analyses

**FIGURE 2:**
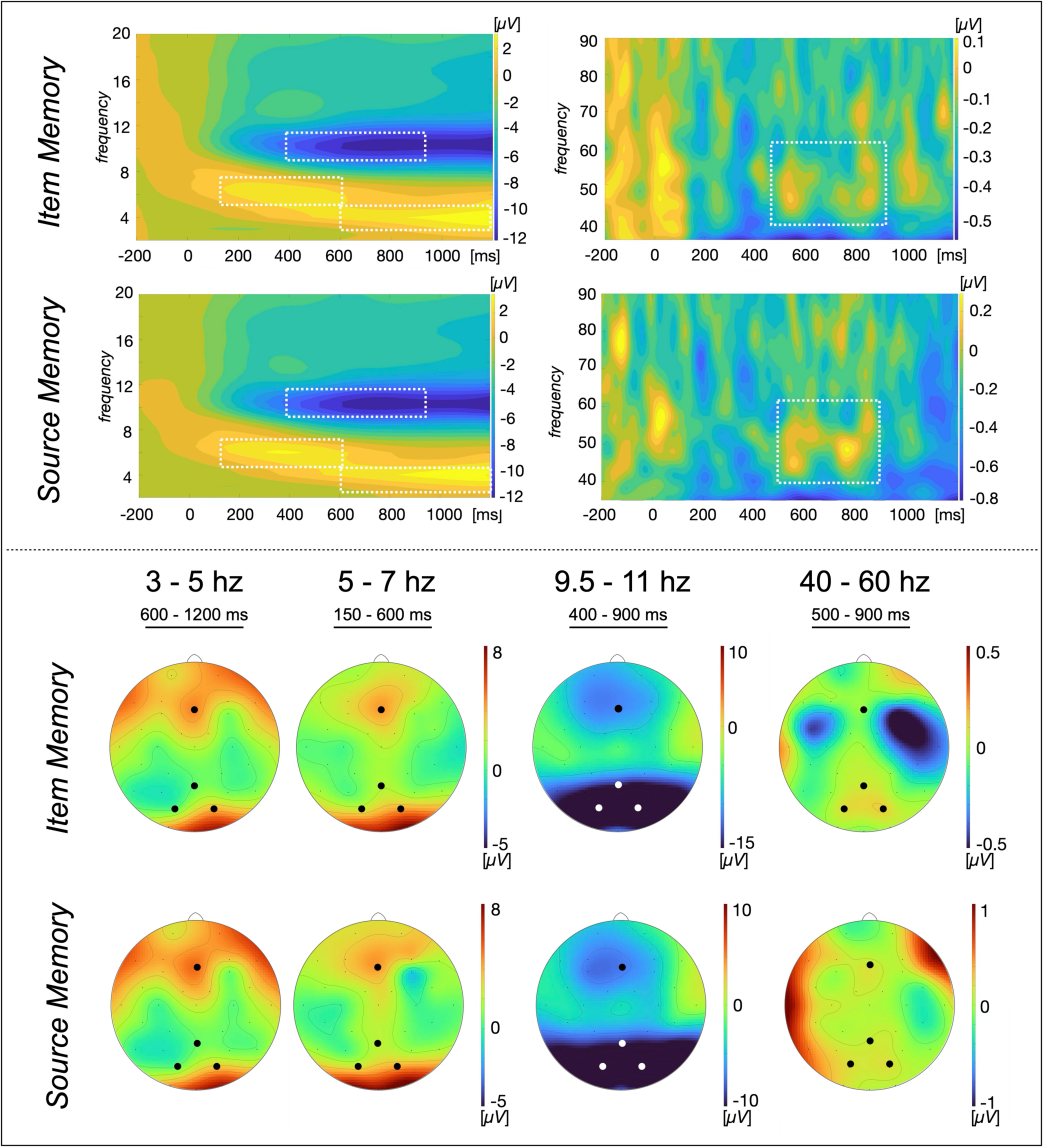
Time‐frequency‐plots and topographies averaged across conditions and modalities. *Note*. The upper panel depicts the time‐frequency (TF)‐plots separately for item and source memory, as well as for the lower (2–20 Hz) and upper (30–90 Hz) frequency ranges to increase visibility. The dotted white lines indicate the frequency ranges and time windows chosen for analyses. The lower panel depicts the topographies averaged across conditions for the chosen time windows and frequencies. The black and white dots indicate the electrodes chosen for analyses (frontal: Fz, parieto‐occipital: Pz, PO4 and PO3). The color of the dots was varied to increase visibility.

For the analyses of the induced frequency response, the raw continuous data was filtered with a 0.25‐Hz high‐pass filter and a 100‐Hz low‐pass filter using the FIR band pass filter (eeglab function). Each electrode was detrended separately. The data were segmented into epochs from 500 ms before, to 1500 ms after stimulus onset, and a baseline correction from −500 ms to stimulus onset was applied. *SCADS* was applied to remove epochs containing blinks (see above). Afterwards, the ICA‐based algorithm for the *correction of saccade‐related transient potentials* (COSTRAP; Hassler et al. 2011) was applied. No remaining flatliners were detected. An ICA was carried out, removing components flagged as eye, muscle, or ECG, respectively (see above). The data were re‐referenced to average reference. A Morlet wavelet analysis was applied to analyze the oscillatory activity. 199 wavelets of seven cycles width were calculated ranging from 1 to 100 Hz. The signal of interest for memory processes was the non‐phase‐locked response, i.e., the induced response, which potentially cancels out in the averaged response as its latency after stimulus onset jitters (Eckhorn et al. 1990). Consequently, the ERP was subtracted from each individual trial before the frequency decomposition was calculated per trial (see Busch et al. 2006). The trials were split into the separate conditions and matched between conditions with respect to the old/new task and the source identification task following the same procedure as for ERP. Participants with fewer than 15 trials per condition after matching were excluded from further analysis (none regarding the old/new task; *n* = 5 for the analyses of the source identification task, including the two participants excluded from LPN‐analysis). The time‐by‐frequency (TF) data was then averaged across the frequency transformations of the single trials per condition (for a similar procedure see Gruber et al. 2008; Kisker, Johnsdorf, Sagehorn, Hofmann, et al. 2025; Kisker, Johnsdorf, Sagehorn, Schöne, et al. 2024). Two regions of interest and thus, two regional means were chosen for analyses based on previous literature, i.e., on the hypotheses: Fz was chosen for the frontal region of interest, and Pz, PO4, and PO3 were averaged regarding the parieto‐occipital region of interest. The time windows of interest were based on previous literature (Gruber et al. 2008) and counterchecked against the time‐frequency (TF)‐plot of the data averaged across all conditions and electrodes. The range of each frequency band was determined based on the TF‐plots as well, resulting in 9.5–11 Hz for the induced ABR and 40–60 Hz for the induced gamma band response (GBR). The induced TBR was subdivided into a lower (3–5 Hz) TBR and upper (5–7 Hz) TBR due to the distinct signatures in the TF‐plot (Figure 2). The electrodes chosen for analyses based on the hypotheses were counterchecked against the regional maxima (see analyses of the *ERP*).

#### 2.5.3 Phase‐Amplitude‐Coupling (PAC)

PAC was determined by means of the modulation index (MI; Tort et al. 2010). The MI relates the phase information of a driving frequency to the amplitude of a signal frequency. Congruently with the spectral power analyses, the PAC was calculated separately for the upper TBR and lower TBR as driving frequencies. In both cases, the gamma band range from 40 to 60 Hz was chosen as the signal frequency. The time window for analysis corresponded to the total time window during which the TBR was analyzed by means of spectral power analysis (150–1200 ms) since longer data epochs allow for more reliable MI calculations. Electrode Fz was chosen for both, driver and signal frequency. We used the same wavelet analyses as for the spectral power analyses to extract the phase of the driver frequency and the amplitude of the signal frequency. The driver frequency's phase was then divided into bins representing different angles. We divided the phase angles into 20 bins, each accounting for 18°. The amplitude of the signal frequency was calculated per bin. Since longer data epochs allow for more reliable MI calculations, the trials were concatenated into one continuous data sequence per subject and condition. Before concatenation, each trial was filtered using a FIR band pass filter (eeglab function). The driving frequency data were filtered with a 1‐Hz high‐pass filter and a 10‐Hz low‐pass filter, while the signal frequency data were filtered with a 30‐Hz high‐pass filter and a 90‐Hz low‐pass filter. A cosine transform was applied to each trial to prevent edge artifacts. The MI was calculated separately for each combination of the driving and signal frequencies before averaging across the given frequency ranges and participants per condition.

### 2.6 Statistical Analyses

#### 2.6.1 Behavioral Data

The parameters operationalizing memory performance (see 2.4) were compared between VR and PC using one‐tailed paired‐sample *t*‐tests. The significance level was Bonferroni‐corrected to 0.013. The participants confidence about their ratings was compared using a 1 × 5 repeated measurement ANOVA (rmANOVA) with the within factor *Condition* (VR‐CS, VR‐FS, PC‐CS, PC‐FS, CR) and followed by two‐tailed paired‐sample *t*‐tests if the rmANOVA indicated a significant effect of *Condition*. The criterion *C* was tested against zero per encoding modality and task, using two‐tailed one‐sample *t*‐tests. Moreover, it was compared between encoding modalities per task using paired‐sample *t*‐tests.

#### 2.6.2 ERP

A rmANOVA with the within‐factor *Condition* was used to analyze the ERP effects. Hence, two 1 × 3 rmANOVA were carried out to analyze the early and late old/new effects, i.e., the FN400 and the parietal LPC. If the rmANOVA indicated a significant effect of *Condition* (VR‐Old, PC‐Old, CR), post hoc *t*‐tests for paired samples were applied. According to the hypotheses, the comparison of both VR‐Old and PC‐Old versus CR was one‐tailed, as well as the comparison between VR‐Old and PC‐Old.

To analyze the LPN effect, a 1 × 5 rmANOVA including the within‐factor *Condition* (VR‐CS, VR‐FS, PC‐CS, PC‐FS, CR) was carried out. Whenever necessary, Greenhouse–Geisser correction was applied. For the post hoc t‐tests, estimates of recollection and familiarity were calculated based on the most current review (Kwon et al. 2023). Stimuli correctly recognized as old and recalled with their correct source were considered to be based more strongly on recollection, i.e., including but going beyond familiarity. Responses to stimuli correctly recognized as old but retrieved with an incorrect source were considered to predominantly reflect familiarity. To isolate the effects of recollection from the proportions attributable to familiarity (i.e., VR‐recollection, PC‐recollection), the response to stimuli whose source was incorrect (FS) was subtracted from the response to stimuli whose source was correct (CS). Congruently, the response to correctly rejected (CR) stimuli was subtracted from the response to stimuli whose source was incorrect (FS) as an estimate of familiarity (i.e., VR‐familiarity, PC‐familiarity). Based on the hypotheses, one‐tailed post hoc *t*‐tests for paired samples were carried out to compare familiarity and recollection effects between and within encoding modalities. The significance threshold for the post hoc *t*‐test was corrected by means of the false discovery rate (FDR) according to the Benjamini‐Hochberg method (Benjamini and Hochberg 1995). The effect sizes partial eta squared (*η* ^*2*^) and Cohen's *d* were calculated. As suggested by a reviewer, non‐significant comparisons were followed up by the calculation of the Bayes Factor (*BF*_10_) using JASP (Kelter 2020).

#### 2.6.3 Frequency Responses

Regarding the old/new task, a 2 × 3 rmANOVA including the within‐factors *Regional Mean* (frontal, parietal) and *Condition* (PC‐Old, VR‐Old, CR) was carried out per frequency range. If it indicated a significant interaction of both factors, a follow‐up rmANOVA was carried out per regional mean containing only the factor *Condition*. This was intended to prevent an accumulation of post hoc *t*‐tests by determining in advance whether only one of the regional means is relevant for the effect. If the follow‐up rmANOVA indicated a significant effect of the factor *Condition* within the respective regional mean, post hoc *t*‐tests for paired samples were applied. Congruent with the ERP analyses, the comparison of both VR‐Old and PC‐Old versus CR were one‐tailed, as well as the comparison between VR‐Old and PC‐Old in case the hypotheses were directed (i.e., for the TBR).

Regarding the source identification task, a 2 × 5 rmANOVA including the within‐factors *Regional Mean* (frontal, parieto‐occipital) and *Condition* (PC‐CS, PC‐FS, VR‐CS, VR‐FS, CR) was carried out per frequency range. In case of a significant interaction of both factors, a 1 × 5 rmANOVA including the factor *Condition* was carried out per *Regional Mean*. Only in case the follow‐up rmANOVA indicated a significant effect of *Condition* within the respective regional mean, post hoc *t*‐tests for paired samples were applied to prevent an accumulation of post hoc *t*‐tests. The comparisons were based on those carried out by Gruber et al. (2008). Whenever necessary, Greenhouse–Geisser correction was applied and the significance threshold for the post hoc *t*‐test was corrected by means of the FDR (Benjamini and Hochberg 1995) and partial eta squared (*η* ^*2*^) and Cohen's *d* were calculated. Congruently with the ERP analyses, non‐significant comparisons were complemented by the calculation of the Bayes Factor (*BF*_10_).

#### 2.6.4 PAC

A rmANOVA with the within‐factors *Driving Frequency* (lower TBR, upper TBR) and *Condition* (PC‐CS, PC‐FS, VR‐CS, VR‐FS, CR) was used to analyze the PAC; i.e., a 2 × 5 rmANOVA was carried out. If the rmANOVA indicated a significant interaction between *Driving Frequency* and *Condition*, it was followed up by a 1 × 5 rmANOVA per driving frequency range containing only the factor *Condition*. Only if this follow‐up rmANOVA indicated an effect of *Condition*, were post hoc *t*‐tests for paired samples applied in accordance with the post hoc comparisons carried out by Gruber et al. (2008). The effect sizes partial eta squared (*η* ^*2*^) and Cohen's *d* were calculated. Non‐significant comparisons were complemented by the calculation of the Bayes Factor (*BF*_10_).

## 3 Results

### 3.1 Encoding Phase

Please refer to Table 1 for an extensive overview of the encoding metrics. Across conditions, participants categorized 156.77 of 200 objects correctly (SD = 31.48). In more detail, participants correctly categorized 76.87 objects in the VR‐condition (SD = 17.00) and 79.90 in the PC‐condition (SD = 15.21). The overall reaction time was 1329.60 ms (SD = 1329.10 ms), with slightly higher response times in the PC‐condition (*M* = 1471.50 ms, SD = 1824.00 ms) and slightly shorter response times in the VR‐condition (*M* = 1187.70 ms, SD = 440.18 ms).

**TABLE 1:** Performance and response time metrics across encoding conditions.

Metric	Overall (*M,* SD)	VR (*M,* SD)	PC (*M,* SD)
Number of correct answers	156.77 (31.48)	76.87 (17.00)	79.90 (15.21)
Response time (ms)	1329.60 (1329.10)	1187.70 (440.18)	1471.50 (1824.00)
Response time for incorrect responses (ms)	1602.20 (1827.80)	1345.10 (535.44)	1859.40 (2523.90)
Response time for correct responses (ms)	1057.00 (263.91)	1030.30 (236.80)	1083.70 (289.94)

*Note:* All values are expressed as mean (SD). No statistical comparisons were carried out. The absolute number of possible correct answers was 200 across both conditions, i.e., 100 per condition.

### 3.2 Behavioral Memory Performance

The memory performance did not differ depending on encoding modality. Participants identified approx. 75% of old stimuli correctly independent of encoding modality (*t*[30] = −0.34, *p* = 0.369, *BF*_10_ = 0.20; *M*_VR_ = 74.3%; *M*_PC_ = 74.8%). 87.92% of the new items were correctly rejected. Of the source identifications made, approx. 61% were correct, independent of the encoding modality (*t*[30] = 0.19, *p* = 0.425, *BF*_10_ = 0.20, *M*_VR_ = 61.8%; *M*_PC_ = 61.0%). Participants' confidence about their ratings did not differ significantly between conditions (*F*[2.14, 64.31] = 1.89, *p* = 0.157, *BF*_10_ = 0.34). On average, they indicated to be sure (rather than unsure or very sure) about their ratings (*M* = 2.79; *M*_PC‐CS_ = 2.83, *M*_PC‐FS_ = 2.79, *M*_PC‐CS_ = 2.65, *M*_PC‐FS_ = 2.89, *M*_PC‐FS_ = 2.79). Last but not least, *d*‐prime indicated a relatively high accuracy in differentiating both VR and PC stimuli from new ones but revealed no significant differences between encoding modalities (*t*[30] = −0.41, *p* = 0.341, *BF*_10_ = 0.20, *M*_VR_ = 1.94; *M*_PC_ = 1.95).

Criterion *C* indicated no significant response bias regarding the old/new task for either encoding modality (*t*_*VR*_ [30] = −0.001, *p* = 0.999, *BF*_10_ = 0.19, *M*_*VR*_ < −0.001, *SD*_*VR*_ = 0.81; *t*_*PC*_ [30] = 0.001, *p* = 0.999; *BF*_10_ = 0.19, *M*_*PC*_ < 0.001, *SD*_*PC*_ = 0.82), as well as regarding the source identification task (*t*_*VRSource*_ [30] = −0.315, *p* = 0.755, *BF*_10_ = 0.20, *M*_*VRSource*_ = −0.03, *SDVR*_*Source*_ = 0.53; *t*_*PCSource*_ [30] = −0.003, *p* = 0.998, *BF*_10_ = 0.19; *M*_*PCSource*_ < 0.001, *SD*_*PCSource*_ = 0.59). Hence, no indication that participants tended to give a certain response when uncertain was found regarding either task. Furthermore, the comparison of both encoding conditions revealed no significant differences (old/new task: *t*[30] = −0.004, *p* = 0.997, *BF*_10_ = 0.19; source identification task: *t*[30] = −0.387, *p* = 0.701, *BF*_10_ = 0.20), indicating that neither encoding condition was significantly more biased than the other.

### 3.3 ERPs

#### 3.3.1 Old/New Task

**FIGURE 3:**
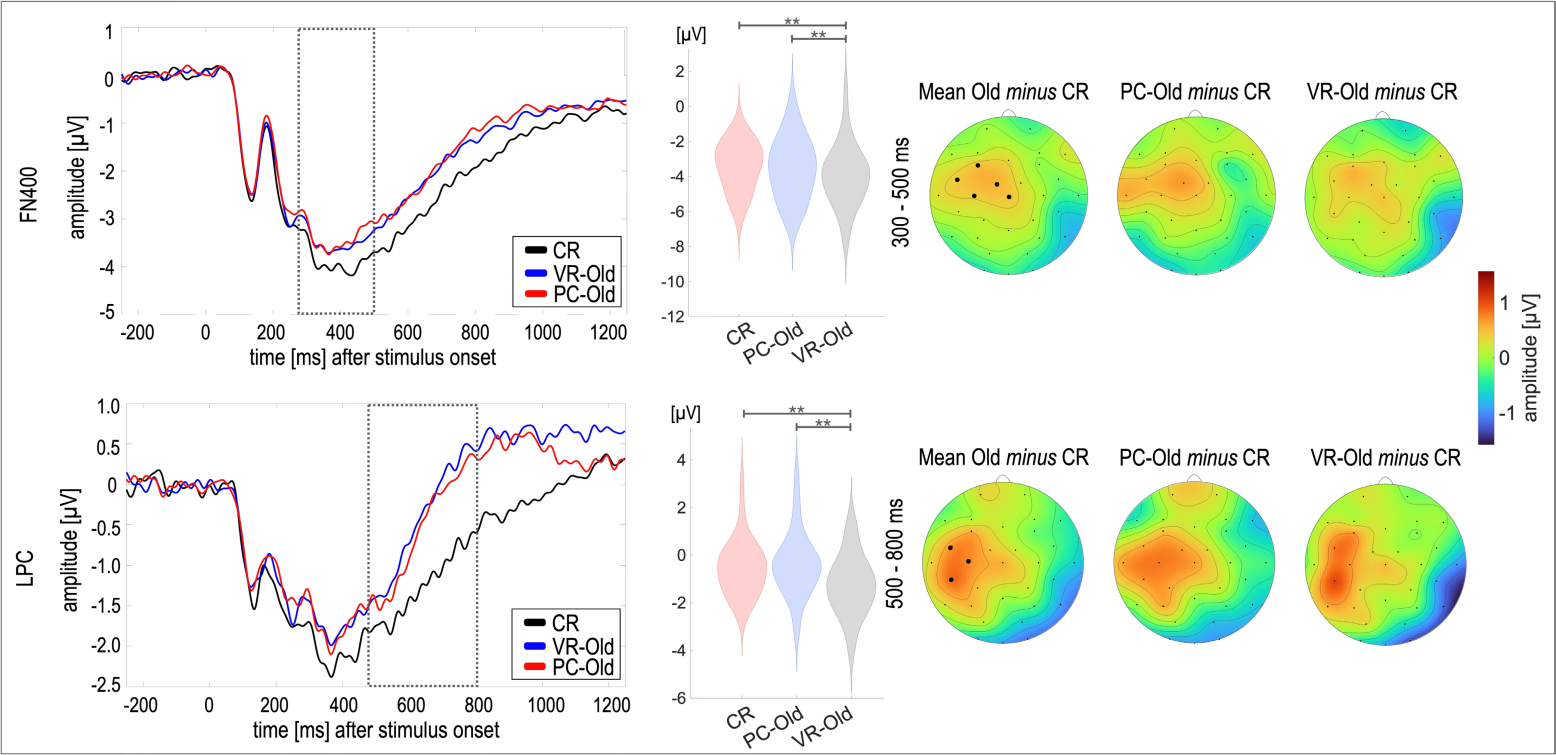
Topography and line plots of the old/new effects. *Note.* The dotted lines in the line plots indicate the time windows for analyses and the black dots in the topographies indicate the electrodes averaged for statistical comparison of the conditions (FN400: F3, FC1, FC5, C3 and Cz; LPC: FC5, C3 and CP5). The violin plots depict the distribution of the data per condition for the electrodes and time window chosen for analyses. The mean topography was calculated as *mean (VR‐Old, PC‐Old) minus CR*. CR = Correct rejection.

Significant effects of *Condition* were observed for both the early, frontally distributed old/new effect (FN400, *F*[2, 60] = 6.15, *p* = 0.004, *η* ^*2*^ = 0.17) as well as for the later, more parietal old/new effect (LPC, *F*[2, 60] = 8.37, *p* < 0.001, *η* ^*2*^ = 0.22). In both cases, classical old/new effects were found, revealing a less negative, i.e., more positive‐going response to old, i.e., VR‐Old and PC‐Old stimuli compared to correct rejections (CR; all *t*s > 2.85, all *p*s < 0.005; *d*s = [0.51.86]; see Figure 3). However, no difference was observed between the responses to VR‐Old and PC‐Old (FN400: *t*[30] = −0.64, *p* = 0.263; LPC: *t*[30] = 0.59, *p* = 0.287). The Bayes Factor (BF) provided moderate evidence for the H_0_ for both comparisons (FN400: *BF*_10_ = 0.232; LPC: *BF*_10_ = 0.225).

#### 3.3.2 Source Identification Task

**FIGURE 4:**
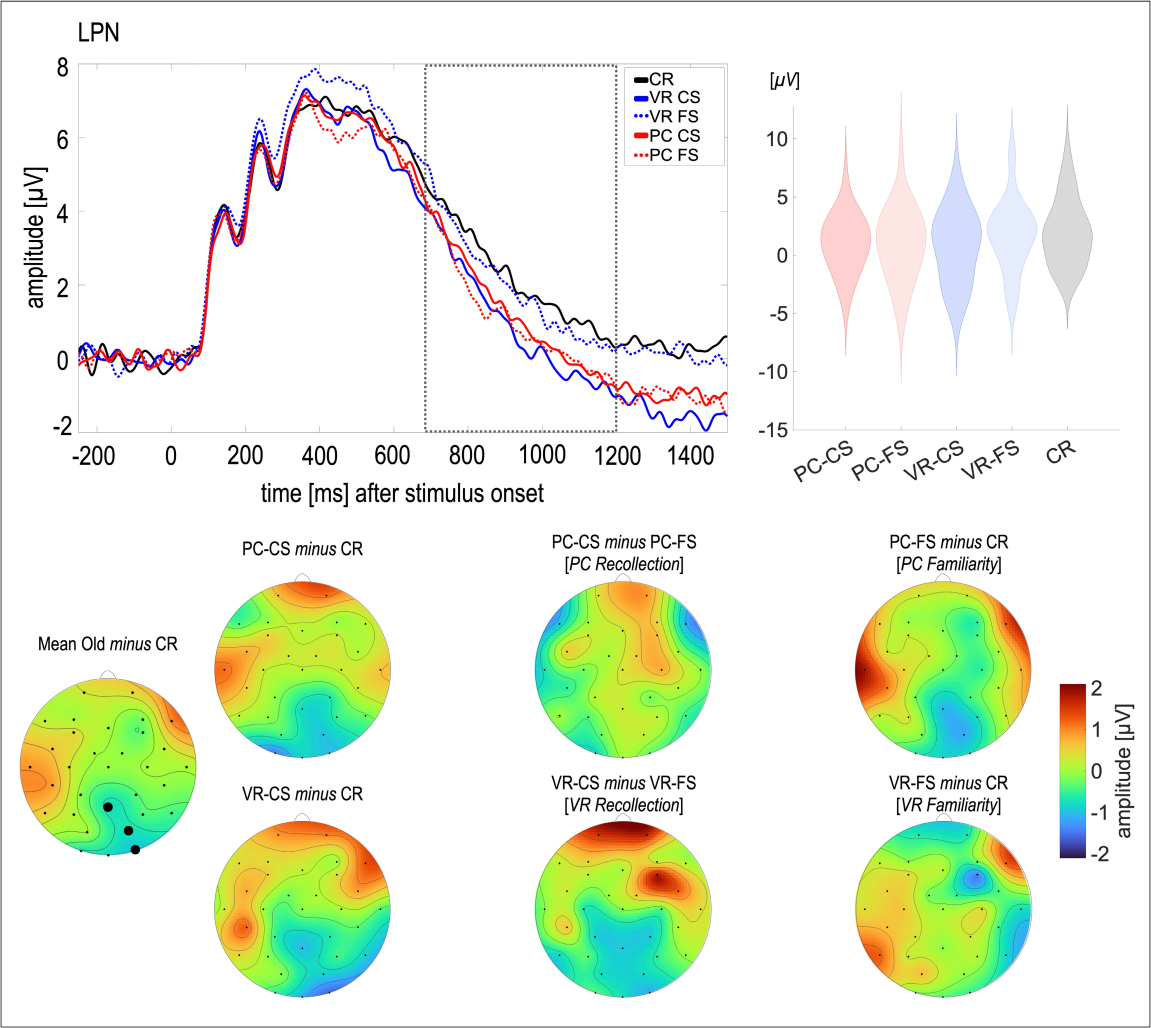
Topography and line plots of the source memory effect. *Note.* The dotted line in the line plot indicates the time window for analysis and the black dots in the leftmost topography indicate the electrodes chosen for analyses (Pz, O2, PO4). The violin plots depict the distribution of the data per condition for the electrodes and time window chosen for analyses. The mean topography was calculated as *mean (VR‐CS, VR‐FS, PC‐CS, PC‐FS) minus CR*. No direct comparisons were marked in the violin plots as in the other graphs, since the estimates based on differences between the conditions were tested against each other. VR‐CS = VR‐encoded item with correct source identification, VR‐FS = VR‐encoded item with false source identification, PC‐CS = PC‐encoded item with correct source identification, PC‐FS = PC‐encoded item with false source identification, CR = Correct rejection.

A significant effect of *Condition* on the LPN was found (*F*[4, 112] = 3.73, *p* = 0.007, *η* ^*2*^ = 0.12). The estimate of recollection (*t*[28] = −2.07, *p* = 0.024, *d* = −0.38; M_VR‐Recollection_ = −1.02, M_PC‐Recollection_ = 0.18) as well as the estimate of familiarity (*t*[28] = 2.38, *p* = 0.012, *d* = 0.44; M_VR‐familiarity_ = −0.28, M_PC‐familiarity_ = −1.17) differed between VR and PC. In particular, the difference between false source identifications and correct rejections, i.e., familiarity, was more negative for PC compared to VR (see Figure 4), while the difference between correct and false source identifications, i.e., recollection, was more negative for VR compared to PC. Counterintuitively, the estimates of recollection and familiarity did not differ significantly within VR (*t*[28] = 0.92, *p* = 0.183, *BF*_10_ = 0.29) but only within PC (*t*[28] = −2.70, *p* = 0.006, *d* = −0.50).

### 3.4 Frequency Band Responses

#### 3.4.1 Old/New Task

**FIGURE 5:**
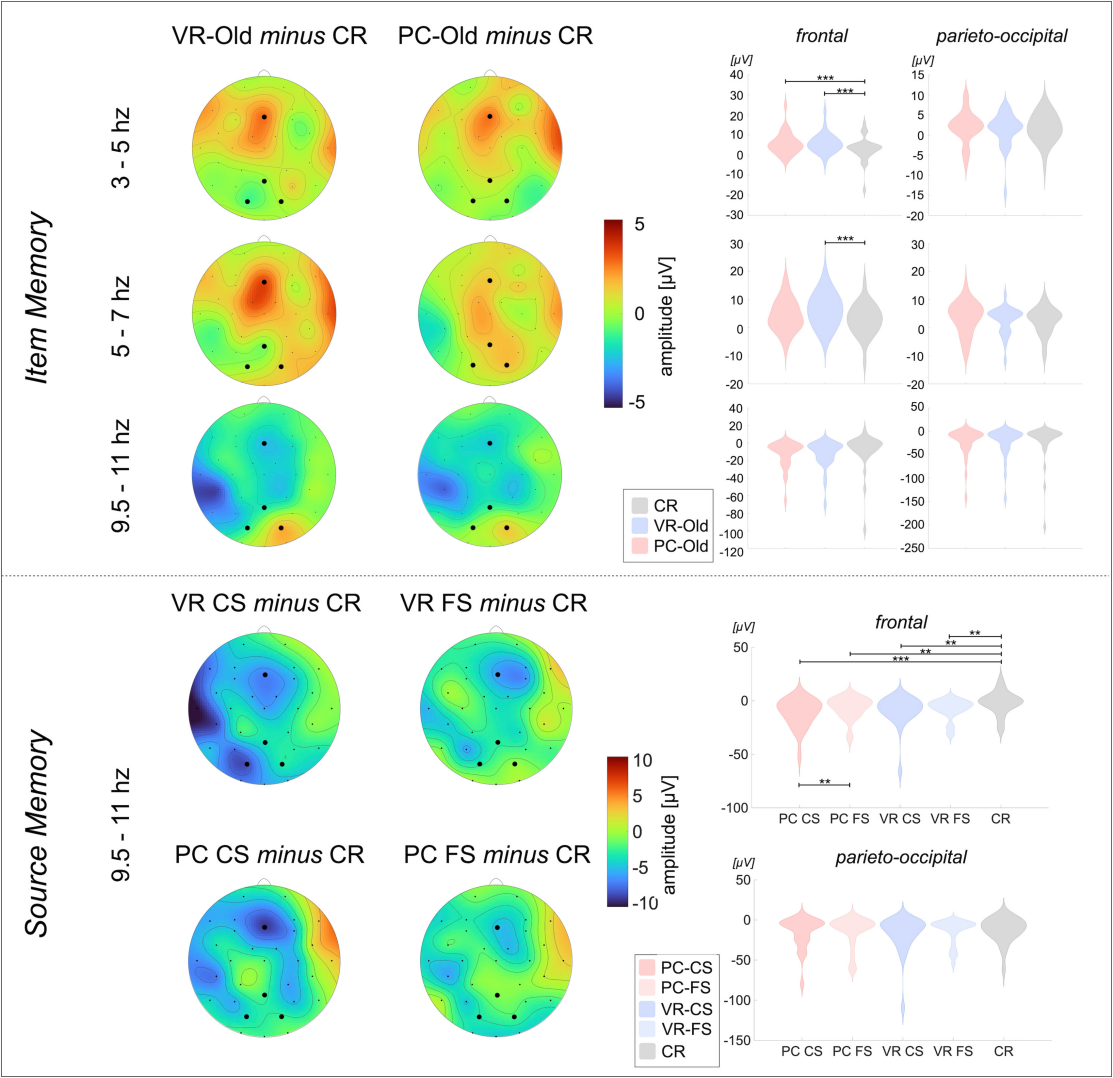
Visualization of the induced oscillatory responses. *Note*. The amplitude distribution is depicted separately for each frequency band range yielding significant effects in the *rmANOVA* and separately for item memory and source memory. The electrodes used for statistical comparisons are marked in the respective mean topographies as black dots (frontal: Fz, parieto‐occipital: Pz, PO4 and PO3). The violin plots indicate each conditions distribution. VR‐CS = VR‐encoded item with correct source identification, VR‐FS = VR‐encoded item with false source identification, PC‐CS = PC‐encoded item with correct source identification, PC‐FS = PC‐encoded item with false source identification, CR = Correct rejection. Comparisons significant after *fdr*‐correction are indicated as follows: *p < 0.05, **p ≤ 0.01, ***p ≤ 0.001.

Regarding the lower TBR and upper TBR, significant main effects of *Regional Mean* (lower TBR: *F*[1, 30] = 21.96, *p* < 0.001, *η* ^*2*^ = 0.42; upper TBR: *F*[1, 30] = 6.80, *p* = 0.014, *η* ^*2*^ = 0.19), *Condition* (lower TBR: *F*[2, 60] = 4.21, *p* = 0.020, *η* ^*2*^ = 0.12; upper TBR: *F*[2, 60] = 3.68, *p* = 0.031, *η* ^*2*^ = 0.11), and their significant interaction (lower TBR: *F*[2, 60] = 8.91, *p* < 0.001, *η* ^*2*^ = 0.34; upper TBR: *F*[1.6, 48.03] = 9.59, *p* < 0.001, *η* ^*2*^ = 0.24) were found. Regarding the ABR, the rmANOVA indicated a significant main effect of *Regional Mean* (*F*[1, 30] = 10.96, *p* = 0.002, *η* ^*2*^ = 0.27) but not of *Condition* (*F*[1.16, 34.70] = 1.03, *p* = 0.330). Yet, a significant interaction of both was found (*F*[1.68, 50.44] = 5.23, *p* = 0.011, *η* ^*2*^ = 0.15). Regarding the GBR, a significant effect of *Regional Mean* (*F*[1, 30] = 7.08, *p* = 0.012, *η* ^*2*^ = 0.19) was observed but no further effects were found (both *F*s < 1.50, both *p*s > 0.20). As differences between the regional means averaged across conditions do not contribute to the research question, no post hoc tests were conducted on the basis of this main effect. The follow‐up rmANOVA per regional mean for upper TBR, lower TBR, and ABR indicated main effects of *Condition* regarding the frontal regional mean (all *F*s > 4.6 all *p*s < 0.025) but not the parieto‐occipital regional mean (all *F*s < 2.0, all *p*s > 0.05). Please find the detailed statistics on the rmANOVA and the post hoc *t*‐Tests in Table 2. In summary, a significantly higher lower TBR was observed in response to PC‐Old compared to CR, as well as to VR‐Old compared to CR (all *t*s >|2.1|, all *p*s < 0.02), whereas the upper TBR revealed a significantly higher response to VR‐Old compared to CR but not to PC‐Old (VR‐Old vs. CR: *t*[30] = 3.55, *p* < 0.001; PC‐Old vs. CR: *t*[30] = 1.57, *p* = 0.064). The ABR did not differ significantly between conditions after FDR correction (all *t*s <|2.5|, all *p*s > 0.017) and the frequency responses to VR‐Old and PC‐Old did not differ significantly across all frequency bands (see Table 2 & Figure 5).

**TABLE 2:** Test statistics for the frequency responses to the old/new task and source identification task.

*Old/new task: rmANOVA*							
Frequency	Regional mean	*df1*	*df2*	*F*	*p*	*η* ^2^	
Lower theta	Frontal	1.61	48.42	7.87	0.002	0.21	
	Parieto‐occipital	2	60	0.59	0.557	0.02	
Upper theta	Frontal	2	60	6.55	0.003	0.18	
	Parieto‐occipital	2	60	1.96	0.150	0.06	
Alpha	Frontal	1.38	41.42	4.70	0.025	0.14	
	Parieto‐occipital	1.14	34.18	0.06	0.841	< 0.01	
***Old/new task: Post hoc paired‐samples t‐tests***							
**Frequency**	**Comparison**	***t*(30)**	**Tail**	**FDR**	***p***	***d***	***BF*_10_**
Frontal lower theta	PC‐Old vs. CR	2.89	One	≤ 0.007	0.004	0.52	
	VR‐Old vs. CR	3.33	One		0.001	0.60	
	VR‐Old vs. PC‐Old	−0.16	One		0.436	−0.03	0.194
Frontal upper theta	PC‐Old vs. CR	1.57	One	≤ 0.001	0.064	0.28	0.576
	VR‐Old vs. CR	3.55	One		0.001	0.64	
	VR‐Old vs. PC‐Old	−2.05	One		0.025	−0.37	1.192
Frontal alpha	PC‐Old vs. CR	−2.10	Two	< 0.017	0.044	−0.38	1.308
	VR‐Old vs. CR	−2.50	Two		0.018	−0.45	2.726
	VR‐Old vs. PC‐Old	0.45	Two		0.659	0.08	0.210
***Source identification task: rmANOVA***							
**Frequency**	**Regional mean**	***df1***	***df2***	***F***	***p***	***η* ^2^**	
Alpha	Frontal	4	25	5.509	< 0.001	0.181	
	Parieto‐occipital	2.84	71.01	1.648	0.188	0.062	
***Source identification task: Post hoc paired‐samples t‐tests***							
**Frequency**	**Comparison**	***t*(25)**	**Tail**	**FDR**	***p***	***d***	***BF*_10_**
Frontal alpha	PC‐CS vs. CR	−3.73	Two	≤ 0.024	< 0.001	−0.73	
	PC‐FS vs. CR	−2.40	Two		0.024	−0.47	
	PC‐CS vs. PC‐FS	−2.48	Two		0.020	−0.49	
	VR‐CS vs. CR	−3.16	Two		0.004	−0.62	
	VR‐FS vs. CR	−3.03	Two		0.006	−0.59	
	VR‐CS vs. VR‐FS	−0.47	Two		0.640	−0.09	0.230

*Note:* The post hoc *t*‐tests were corrected by means of the false discovery rate (FDR; Benjamini and Hochberg 1995). The column *FDR* thus indicates the FDR‐corrected threshold for significance. Whether *t*‐tests were carried out one‐ or two‐tailed was chosen based on the hypotheses (see Section 2.6). The Bayes factor was calculated as a complement for non‐significant comparisons.

#### 3.4.2 Source Identification Task

Regarding the upper TBR and GBR, no significant main effects or interactions were found (see Table 2). For the lower TBR, a main effect of *Regional Mean* was found (*F*[1, 30] = 11.50, *p* = 0.002, *η* ^*2*^ = 0.32), which was not followed up by post hoc tests since the difference between regional means averaged across conditions is not informative for the research question at hand. Yet significant main effects of *Regional Mean* (*F*[1, 25] = 16.05, *p* < 0.001, *η* ^*2*^ = 0.39), *Condition* (*F*[4, 100] = 3.54, *p* = 0.008, *η* ^*2*^ = 0.13), and a significant interaction of both (*F*[4, 100] = 3.54, *p* = 0.010, *η* ^*2*^ = 0.12) were found regarding the ABR. The follow‐up rmANOVA per regional mean for the ABR indicated a main effect of *Condition* regarding the frontal regional mean (*F*[4,100] = 5.51, *p* < 0.001, *η* ^*2*^ = 0.18) but not the parieto‐occipital regional mean (*F*[4,100] = 1.65, *p* = 0.168). The subsequent post hoc *t*‐tests revealed significantly more negative ABR to PC‐CS, PC‐FS, VR‐CS, and VR‐FS compared to CR, respectively (all *t*s >|2.40|, all *p*s < 0.024). Yet while the ABR to PC‐CS was significantly more negative than to PC‐FS (*t*[25] = −2.48, *p* = 0.02), no significant difference was found between VR‐CS and VR‐FS (*t*[25] = −0.47, *p* = 0.64.). Please refer to Table 1 for the detailed statistics.

### 3.5 PAC

**FIGURE 6:**
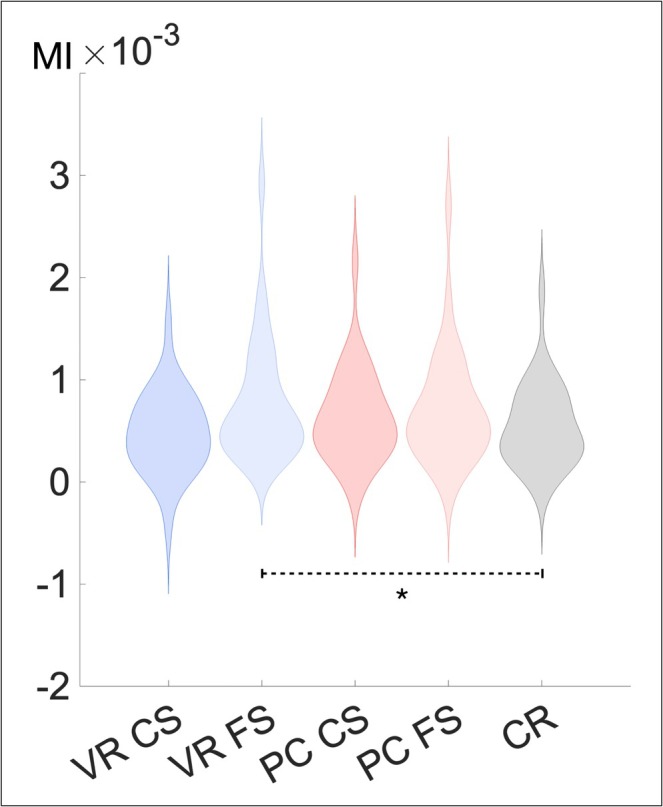
Violin plots of the modulation index per condition. *Note*. Violin plots of the baseline corrected average MI for each condition, indicating the PAC between the lower TBR and the GBR. Significance of differences between conditions are marked as: *p < 0.05. VR‐CS = VR‐encoded item with correct source identification, VR‐FS = VR‐encoded item with false source identification, PC‐CS = PC‐encoded item with correct source identification, PC‐FS = PC‐encoded item with false source identification, CR = Correct rejection.

A significant main effect of the factor *Driving Frequency* (*F*[1, 28] = 43.22, *p* < 0.001, *η* ^*2*^ = 0.61) and an interaction with the factor *Condition* (*F*[4, 112] = 3.19, *p* = 0.016, *η* ^*2*^ = 0.10) was found, whereas no main effect of *Condition* was observed (*F*[3.12, 87,31] = 1.62, *p* > 0.10). The follow‐up rmANOVA revealed an effect of *Condition* for the lower TBR as driving frequency (*F*[4, 112] = 3.06, *p* = 0.019, *η* ^*2*^ = 0.10) but not for the upper TBR (*F*[4, 112] = 0.40, *p* = 0.81). Post hoc *t*‐tests based on the lower TBR as driving frequency revealed a significantly higher PAC in the VR‐FS condition compared to CR (*t*[28] = 2.22, *p* = 0.035, *d* = 0.41; see Figure 6). The comparisons between VR‐CS, PC‐CS, and PC‐FS with CR did not reach significance (all *t*s <|1.65|, all *p*s > 0.10, all *BF*_*10*_ < 0.63).

## 4 Discussion

The study examined whether the retrieval of VR‐engrams is more profoundly based on recollection and less on familiarity compared to PC‐engrams, and whether differences in source memory contribute to differences in retrieval processes of VR‐ and PC‐engrams reported in previous studies. Crucially, our study aimed to provide a foundational understanding for whether and to what extent the encoding modality (i.e., VR versus PC) serves as a relevant source for recollection. This understanding offers a baseline for subsequent differentiations of the extent to which further context information beyond the encoding modality affects retrieval processes. To this end, participants incidentally encoded everyday objects in VR and on a conventional desktop (PC). In a following unannounced recognition task, they were asked to indicate whether they remembered the objects from the encoding phase or not (old/new task; item memory), and in case of old decisions, whether they encoded it in VR or on desktop (source identification task; source memory). The behavioral markers of memory performance and confidence, as well as the electrophysiological markers linked to item memory, i.e., the FN400, the parietal LPC, and the TBR, yielded comparable results for both encoding modalities. Yet the electrophysiological correlates linked to source memory, particularly the LPN, indicated a shift in retrieval processes depending on the encoding modality.

Adding to previous, rather heterogeneous findings, our results revealed no superior retrieval performance regarding engrams obtained from the VR‐based condition (hereafter: VR‐engrams) compared to the engrams obtained from the desktop‐based condition (hereafter: PC‐engrams). Participants correctly identified 75% of old stimuli in the old/new task irrespective of the encoding modality, as well reflected in relatively high *d*‐prime values and subjective confidence about their ratings. Moreover, 61% of all source identifications of either encoding modality were correct, indicating that this task was more difficult compared to the old/new task but above chance level. A response bias was controlled for as an alternative explanation since Criterion *C* neither indicated a response bias for either task nor between encoding modalities. Hence, the observed results cannot be traced back to a tendency in the response behavior. Consequently, our results point to two possible indications: On the one hand, our results suggest that three‐dimensionality through the use of a VR headset alone does not facilitate increased behavioral memory performance compared to conventional desktop conditions. In particular, we matched the conditions beyond dimensionality, varying only 2D‐ and 3D‐presentation between the two conditions. The visual context, the way of stimulus presentation, and the extent of possible interactions were maintained across conditions as well. In contrast, previous studies reporting superior retrieval of VR‐engrams varied more rigorously between conditions, e.g., in haptic features (Schöne et al. 2019), complex contexts by means of photorealistic scenes (Schöne et al. 2019, 2021), or interactive, explorable environments (Harman et al. 2017; Krokos et al. 2019). Consequently, the change from 2D to 3D presentation alone is not associated with significant increases in memory performance at the behavioral level. This result provides a starting point to disentangle potential effects of further immersive features on memory performance.

Yet on the other hand, VR‐engrams were equally well remembered compared to PC‐engrams *despite* a potential disadvantage due to a more considerable change between encoding context and retrieval context. The well‐established encoding‐specificity hypothesis (Tulving and Thomson 1973) proposes that memory performance and overlap between encoding and retrieval contexts are positively related. In other words, the greater the match between both contexts, the better the performance of memory retrieval (Godden and Baddeley 1975; Tulving and Thomson 1973), which has been replicated for experimental setups based on VR as well (Shin et al. 2021). In our study, both conditions included a change of context between encoding and retrieval. However, the change of context went further regarding the VR‐condition, since the stimuli were presented in 3D during encoding and 2D during retrieval. The non‐significant difference in memory performance for VR‐ and PC‐engrams thus suggests that the VR‐engrams were remembered well enough to compensate for potentially stronger context effects. Accordingly, a next important and informative step would be to investigate whether memory performance varies or not when both kinds of engrams are retrieved under VR‐conditions instead of PC‐conditions.

Congruent with the behavioral data, the ERP correlates of item memory indicate that encoding modality by means of presenting stimuli either two‐ or three‐dimensionally while matching further contextual and interactive features does not shift the retrieval processes and performance significantly. Irrespective of the encoding modality, the FN400 related to familiarity and the parietal LPC related to recollection yielded a more positive response, i.e., less negative response, to old stimuli compared to correctly rejected distractors. Similarly, the lower TBR did not differentiate between VR‐ and PC‐engrams, reflecting the conventional old/new effect independent of the encoding modality. However, the upper TBR differentiated only VR‐engrams from new stimuli but neither one from PC‐engrams. Both the conventional old/new effect in the lower TBR, as well as the VR‐specific effect in the upper TBR are in stark contrast to a previous study comparing the theta old/new effect between VR‐engrams and PC‐engrams, reporting this effect for PC‐engrams but not VR‐engrams (Kisker et al. 2021b). While the lower (or slower) TBR has been more strongly associated with recollection and conscious awareness, the upper (or faster) TBR has been linked to interference resolution (Pastötter and Bäuml 2014). Consequently, these results indicate that the retrieval of VR‐engrams exhibited processes to overcome interferences, e.g., inhibiting irrelevant memories going beyond the retrieval of PC‐engrams (Hanslmayr et al. 2010; Pastötter and Bäuml 2014).

Moreover, VR‐engrams yielded similar attentional processing demands compared to PC‐engrams with respect to item memory, which was reflected in the ABR; whereas the previous study reported lower attentional demands for VR‐engrams (Kisker et al. 2021b). While this pattern of results was previously interpreted as indicating differences in retrieval effort (Kisker et al. 2021b), our current study indicates no such difference. In synthesis, these results might very well be explained by the stringent match of the encoding conditions beyond dimensionality, thereby maintaining effortful control processes related to recollection at consistent levels across both conditions regarding the lower TBR. In contrast, using photorealistic, 3D‐360° videos with spatial sound might shift these processes more strongly across conditions. Independent of the immersiveness of either VR condition, the retrieval of complex scenes might exhibit processes distinct from the retrieval of isolated everyday objects (for a review on scene processing, see e.g., Castelhano and Krzyś 2020).

Putting the results with respect to item memory in a nutshell, they provide an essential understanding allowing for subsequent differentiation of how additional information from the encoding episode beyond modality affects retrieval processes. In other words, determining the effects of the presentation modality on retrieval was an indispensable, previously omitted step to isolate effects of modality and stimulus content.

Yet even more importantly, our results regarding source memory indicate meaningful differences in familiarity and recollection depending on the encoding modality. Based on the LPN's association with the amount of context information available during retrieval (Johansson and Mecklinger 2003; Mecklinger et al. 2007, 2016), recollection was renormalized as the difference between correct source judgments and incorrect source judgments, while familiarity was quantified as the difference between incorrect source judgments and correct rejections (Kwon et al. 2023). In contrast to the parietal LPC, yet in line with the hypotheses, the LPN‐based estimate of recollection differed between VR and PC, indicating higher recollection in the VR condition. The estimate of familiarity further informs this shift of retrieval processes, indicating less familiarity for VR‐engrams compared to PC‐engrams. Both findings are remarkable considering that the markers related to item memory did not indicate differences beyond an old/new effect in the upper TBR observed for VR‐engrams only.

In more detail, the difference between false source identifications and correct rejections was larger for PC‐ compared to VR‐engrams, indicating a stronger familiarity‐related effect in the PC condition. In contrast, the response to VR‐engrams with false source retrieval (VR‐FS) was descriptively relatively similar to the response to correctly rejected items, resulting in a low estimate of familiarity. With respect to the functional significance of the LPN, this indicates that only a low amount of context information was available for integration regarding VR‐engrams whose source judgment was incorrect. This indicates that although VR‐engrams resulted in higher recollection compared to PC‐engrams, the in comparison attenuated familiarity may be of greater relevance in cases where only the modality is varied by means of 2D desktop presentation and 3D VR presentation. The attenuated familiarity of VR‐engrams may stem from context transfer effects, as contextual mismatch alters the LPN response even during real‐world object retrieval (Park and Donaldson 2019). The two‐dimensional retrieval cue may better match the two‐dimensional encoding of PC stimuli while resulting in a mismatch for three‐dimensionally encoded VR‐engrams, potentially fostering recollection at the expense of familiarity. However, this interpretation is limited by the insignificant difference between both estimates *within* the VR condition.

Yet this line of thought is further underpinned by the significantly higher PAC between the lower TBR and GBR only for VR‐engrams whose source was falsely recalled compared to correctly rejected items. Although the PAC has been proposed to reflect efficient mnemonic processing by top‐down regulation on cortical object representations (Graetz et al. 2019; Lisman and Jensen 2013), the GBR has recently been found to mirror uncertainty or incorrectness about the retrieval decision (Wynn et al. 2024). The PAC might thus reflect retrieval attempts of uncertain or incorrect contextual information for VR‐engrams retrieved without their correct source. Beyond the PAC, the spectral analysis of the GBR indicated no old/new effect, and the TBR indicated no differentiation with respect to the correctness of the source retrieval (see Gruber et al. 2008). This limitation might result from the limited number of trials resulting from matched trial numbers between five conditions.

Complementing these results, attentional processing of VR‐engrams might less strongly depend on the correctness of source retrieval compared to PC‐engrams. The frontal ABR more strongly desynchronized in response to both kinds of VR‐ and PC‐engrams, thus indicating higher memory load and attentional processes for old stimuli (Klimesch 1999; Sauseng et al. 2009). In line with recollection being a more controlled process, the ABR differed within PC‐engrams depending on the correctness of the retrieved source, with items retrieved with their correct source affording more attentional resources or memory load, i.e., a stronger ABR desynchronization. Yet the ABR did not differ between both kinds of VR‐engrams. Since VR‐engrams yielded less familiarity, both kinds of engrams might afford similar levels of attentional processing for retrieval (Yonelinas 2002).

Overall, VR‐engrams whose source is not correctly remembered, and which are therefore considered familiar but not recollected, occupy a special position across the analyzed measures associated with source memory retrieval. Taken together, these measures suggest that some information is not available regarding these engrams but for PC‐engrams retrieved with the false source. It would thus be plausible, for example, that perceptual 2D cues from the encoding phase that match the retrieval cue are included in PC‐engrams which foster familiarity. Yet these matching cues would not be available for VR‐engrams during retrieval due to the three‐dimensional encoding. This might lead to attenuated familiarity and explain the less negative LPN response to VR‐engrams retrieved with an incorrect source, whereas the LPN did not differentiate PC‐engrams depending on the accuracy of source identification.

Emerging updates on the Dual Process Theory offer an overlapping explanation by extending recognition memory processes to a tri‐component model (Addante et al. 2024). In particular, familiarity is further subdivided into item familiarity and context familiarity, the latter denoting low confidence in the recognition of an item despite correct source retrieval. Most recently, Addante et al. (2024) demonstrated that context familiarity is distinct from item familiarity in recognition, providing evidence of a unique negative‐going, centrally distributed ERP component distinct from the FN400 and LPC effects related to (item) familiarity and recollection, a pattern supported by further neuroimaging studies (Good et al. 2007; Martin et al. 2013). With respect to this framework, VR‐engrams retrieved with an incorrect source might lack context familiarity rather than item familiarity as discussed above in the light of the contextual mismatch between encoding and retrieval. This interpretation is consistent with the observation that, quantified by the LPN, the least amount of context information was available for reconstructing the encoding phase for this condition. However, it touches upon a potential overlap of the LPN and the broad central negativity (BCN) associated with context familiarity. Yet, as argued by Addante et al. (2024) as well as Mecklinger et al. (2016), it is unlikely that the BCN and LPN reflect the same ERP components—in particular, the LPN is characterized by a posterior maximum, whereas the BCN is associated with a centrally distributed maximum. In our data, the amplitude was maximally negative around electrode O2, which corresponds to the posterior distribution of the LPN, whereas positive amplitudes were observed descriptively at central electrodes. Even more importantly, the correlates of context familiarity were found to exhibit neither the FN400 nor the LPC old/new effects (Addante et al. 2024). Since engrams correctly recognized as old and retrieved with a correct source might be retrieved based on context familiarity with respect to the tri‐component approach, we counterchecked whether items retrieved with a correct source would elicit both old/new effects (see supplementary material S1). In summary, engrams retrieved with correct source exhibited the LPC old/new effect, whereas the FN400 effect did not reach significance (see supplementary material S1). However, the Bayes factor for the latter provided only anecdotal evidence for equality of the responses to both kinds of old and correctly rejected stimuli, indicating rather absence of evidence than evidence of absence. Yet, the LPC reaching significance while the FN400 did not corresponds to the link between correct source identification and stronger recollection. Consequently, even though our study does not directly contribute to the disentanglement of the processes underlying the BCN and LPN, respectively (for in‐depth discussions, please refer to Addante et al. 2024; Mecklinger et al. 2016), our results are more consistent with the interpretation of the LPN as a correlate of the amount of available contextual information during retrieval.

## 5 Conclusions

In conclusion, the pattern of results has two overall implications: Firstly, that encoding modality—by means of 2D stimuli on a desktop versus 3D stimuli within an immersive environment—has only a limited impact on the processes underlying item memory retrieval under the premise that the encoding contexts and stimuli are matched beyond dimensionality. The same conclusions about memory performance and the underlying processes would be drawn from the electrophysiological correlates linked to the old/new task. This foundational understanding allows for attributing potential differences to characteristics beyond 3D presentation in a VR headset in subsequent studies which implement further immersive features differentiating the encoding modalities.

Yet secondly, and most importantly, our results indicate that encoding modality functions to some degree as a relevant source for recognition memory even if contextual features beyond mere two‐ or three‐dimensionality are matched. In particular, VR‐based encoding is related to meaningful shifts with respect to source memory retrieval as reflected in the LPN, the ABR, and PAC between lower TBR and GBR. Consequently, our results indicate a shift towards recollection regarding the retrieval of VR‐engrams due to attenuated familiarity when compared to PC‐engrams.

## 6 Limitations and Future Directions

Our study examined whether encoding modality served as a key source memory cue, thereby tying source memory entirely to the presentation mode, with no other source information tested. Potentially task‐irrelevant recollections, so‐called “non‐criterial” recollections (Johnson et al. 1993; Yonelinas and Jacoby 1996), were thus ignored. Participants might, however, generally recall the presentation medium (VR/PC), additional information beyond the medium, or both. Consequently, the accuracy of source retrieval would partly depend on the specific information the task referred to, and might lead to underestimating recollection performance or overestimating familiarity (Parks 2007; Yonelinas and Jacoby 1996). Consequently, we kept contextual information beyond modality equal across conditions. However, examining different kinds of source information in VR‐ versus PC‐based encoding could offer insights into whether the presentation medium or further contextual information is more relevant for source identification, retrieval, and performance. Our future research will thus consider source information beyond presentation modality based on the baseline provided in this study.

Furthermore, the pattern of results hints at context transfer effects attenuating familiarity regarding VR‐engrams. To quantify these effects, an equivalent study should be conducted in which both VR‐ and PC‐engrams are recalled under VR‐conditions. If the effects found in the current study were reversed in a 1:1 manner; i.e., if PC‐engrams with false source identification particularly stood out under VR‐based retrieval conditions, this would underpin that the observed modality effects might be primarily based on context transfer effects, and thus accounted for in future studies.

The electrodes and time windows for the analysis of the LPN were derived from preceding literature. Although our results show the characteristics of the LPN using these parameters, there are further studies investigating a later time window, i.e., 1200–1800 ms, reflecting the maintenance of the retrieved episode (e.g., Herron 2007; see Mecklinger 2000; Mecklinger et al. 2007). This time window was not adequate for our analyses as it is interrupted by the offset of the stimulus and the participants' ratings. In follow‐up studies, the later time window should be additionally considered.

Regarding the spectral analyses, we focused on analyzing the theta, alpha, and gamma band ranges but omitted the beta band range. Yet growing evidence highlights beta oscillations as a key indicator of top‐down control from the prefrontal cortex (PFC) and posterior parietal cortex (PPC) on the medial temporal lobe (MTL) and further areas. In particular, the beta band's strongest relevance lies in working memory (e.g., Antzoulatos and Miller 2016; Brincat and Miller 2015; Lundqvist et al. 2016, 2024; Siegel et al. 2009; Stanley et al. 2018), memory formation processes (e.g., Lundqvist et al. 2024; Stanley et al. 2018), and categorization of information (e.g., Antzoulatos and Miller 2016; Stanley et al. 2018). While recent work extends this to retrieval processes (Das and Menon 2023, 2024), only a few (non‐invasive) EEG studies applying old/new or source identification tasks examine the beta band response as a signature of familiarity and recollection (for exceptions see e.g., Hanslmayr et al. 2012; Khader and Rösler 2011). Based on the valuable suggestion of one reviewer, we included analyses of the beta band response as supplementary material, indicating a left‐parietal old/new effect, i.e., a more negative beta band response to old stimuli compared to correct rejections, for both VR‐ and PC‐engrams. Yet no difference was found depending on the encoding modality (see supplementary material S2). Since this analysis was not planned a priori, we included it as supplementary material to emphasize the importance of considering beta in future analyses of episodic retrieval processes. In our case, however, the results overlap with, and thus do not provide insights beyond the a priori planned frequency analyses.

## Author Contributions

**Joanna Kisker:** conceptualization (equal), methodology (equal), software (lead), investigation (supporting), formal analysis (lead), visualization (lead), writing – original draft (lead), writing – review and editing (lead), project administration (lead). **Marius Soethe:** software (supporting), investigation (equal), formal analysis (supporting), writing – review and editing (supporting). **Jonas Sieverding:** software (supporting), investigation (equal), writing – review and editing (supporting). **Leon Lange:** formal analysis (supporting), writing – review and editing (supporting). **Merle Sagehorn:** writing – review and editing (supporting). **Benjamin Schöne:** writing – review and editing (supporting). **Thomas Gruber:** conceptualization (equal), methodology (equal), supervision (lead), resources (lead), formal analysis (supporting), writing – review and editing (supporting).

## Ethics Statement

The studies involving human participants were reviewed and approved by the local ethic committee of Osnabrück University, Germany (reference: Ethik‐58/2023). The participants provided their written informed consent to participate in this study.

## Conflicts of Interest

The authors declare no conflicts of interest.

## Peer Review

The peer review history for this article is available at https://www.webofscience.com/api/gateway/wos/peer‐review/10.1111/ejn.70239.

## Supporting information


**Figure S1:** Topographical amplitude distribution of the contrasts between engrams with correct source retrieval and correctly rejected items based on the old/new analyses of the FN400 and LPC.
**Figure S2:** Visualization of the induced beta band response.

## Data Availability

The data presented in this study can be found in the online repository OSF: https://osf.io/h7yfz/?view_only=15f406b47eb24c289aa4403aa2061e8f.

## References

[ejn70239-bib-0001] Addante, R. J. , E. Clise , R. Waechter , J. Bengson , D. L. Drane , and J. Perez‐Caban . 2024. “Context Familiarity Is a Third Kind of Episodic Memory Distinct From Item Familiarity and Recollection.” iScience 27, no. 12. 10.1016/j.isci.2024.111439. PMC11699256 39758982

[ejn70239-bib-0002] Addante, R. J. , A. J. Watrous , A. P. Yonelinas , A. D. Ekstrom , and C. Ranganath . 2011. “Prestimulus Theta Activity Predicts Correct Source Memory Retrieval.” Proceedings of the National Academy of Sciences 108, no. 26: 10702–10707. 10.1073/pnas.1014528108 PMC3127901 21670287

[ejn70239-bib-0003] Antzoulatos, E. G. , and E. K. Miller . 2016. “Synchronous Beta Rhythms of Frontoparietal Networks Support Only Behaviorally Relevant Representations.” eLife 5: e17822. 10.7554/eLife.17822. 27841747 PMC5148609

[ejn70239-bib-0004] Benjamini, Y. , and Y. Hochberg . 1995. “Controlling the False Discovery Rate: A Practical and Powerful Approach to Multiple Testing.” Journal of the Royal Statistical Society: Series B: Methodological 57, no. 1: 289–300.

[ejn70239-bib-0005] Bonnail, E. , J. Frommel , E. Lecolinet , S. Huron , and J. Gugenheimer . 2024. “Was it Real or Virtual? Confirming the Occurrence and Explaining Causes of Memory Source Confusion between Reality and Virtual Reality.” In *Proceedings of the 2024 CHI Conference on Human Factors in Computing Systems*, 1–17.

[ejn70239-bib-0006] Brincat, S. L. , and E. K. Miller . 2015. “Frequency‐Specific Hippocampal‐Prefrontal Interactions During Associative Learning.” Nature Neuroscience 18, no. 4: 576–581. 10.1038/nn.3954. 25706471 PMC4444366

[ejn70239-bib-0007] Bröder, A. , and T. Meiser . 2007. “Measuring Source Memory.” Zeitschrift Für Psychologie/Journal of Psychology 215, no. 1: 52–60.

[ejn70239-bib-0008] Busch, N. A. , C. S. Herrmann , M. M. Müller , D. Lenz , and T. Gruber . 2006. “A Cross‐Laboratory Study of Event‐Related Gamma Activity in a Standard Object Recognition Paradigm.” NeuroImage 33, no. 4: 1169–1177. 10.1016/j.neuroimage.2006.07.034. 17023180

[ejn70239-bib-0009] Cadet, L. B. , and H. Chainay . 2020. “Memory of Virtual Experiences: Role of Immersion, Emotion and Sense of Presence.” International Journal of Human Computer Studies 144, no. February: 102506. 10.1016/j.ijhcs.2020.102506.

[ejn70239-bib-0010] Castelhano, M. S. , and K. Krzyś . 2020. “Rethinking Space: A Review of Perception, Attention, and Memory in Scene Processing.” Annual Review of Vision Science 6, no. 1: 563–586. 10.1146/annurev-vision-121219-081745 32491961

[ejn70239-bib-0011] Clayton, M. S. , N. Yeung , and R. Cohen Kadosh . 2018. “The Many Characters of Visual Alpha Oscillations.” European Journal of Neuroscience 48, no. 7: 2498–2508. 10.1111/ejn.13747. 29044823

[ejn70239-bib-0012] Cohen, J. 1988. “Statistical Power Analysis for the Behavioral Sciences.” In Statistical Power Analysis for the Behavioral Sciences, 2nd ed. Hillsdale, NJ: Lawrence Erlbaum Assoc.

[ejn70239-bib-0013] Cycowicz, Y. M. , D. Friedman , and J. G. Snodgrass . 2001. “Remembering the Color of Objects: An ERP Investigation of Source Memory.” Cerebral Cortex 11, no. 4: 322–334. 11278195 10.1093/cercor/11.4.322

[ejn70239-bib-0014] Das, A. , and V. Menon . 2023. “Concurrent‐ and After‐Effects of Medial Temporal Lobe Stimulation on Directed Information Flow to and From Prefrontal and Parietal Cortices During Memory Formation.” Journal of Neuroscience 43, no. 17: 3159–3175. 10.1523/JNEUROSCI.1728-22.2023. 36963847 PMC10146497

[ejn70239-bib-0015] Das, A. , and V. Menon . 2024. “Frequency‐Specific Directed Connectivity Between the Hippocampus and Parietal Cortex During Verbal and Spatial Episodic Memory: An Intracranial EEG Replication.” Cerebral Cortex 34, no. 7: bhae287. 10.1093/cercor/bhae287. 39042030 PMC11264422

[ejn70239-bib-0016] Delorme, A. , and S. Makeig . 2004. “EEGLAB: An Open Source Toolbox for Analysis of Single‐Trial EEG Dynamics Including Independent Component Analysis.” Journal of Neuroscience Methods 134, no. 1: 9–21. 15102499 10.1016/j.jneumeth.2003.10.009

[ejn70239-bib-0017] Downs, L. , A. Francis , N. Koenig , et al. 2022. “Google Scanned Objects: A High‐Quality Dataset of 3D Scanned Household Items.” In *2022 International Conference on Robotics and Automation (ICRA)*, 2553–2560. IEEE.

[ejn70239-bib-0018] Eckhorn, R. , H. J. Reitboeck , M. T. Arndt , and P. Dicke . 1990. “Feature Linking via Synchronization Among Distributed Assemblies: Simulations of Results From Cat Visual Cortex.” Neural Computation 2, no. 3: 293–307.

[ejn70239-bib-0019] Ernstsen, J. , S. C. Mallam , and S. Nazir . 2019. “Incidental Memory Recall in Virtual Reality: An Empirical Investigation.” Proceedings of the Human Factors and Ergonomics Society Annual Meeting 63, no. 1: 2277–2281. 10.1177/1071181319631411.

[ejn70239-bib-0020] Faul, F. , E. Erdfelder , A. G. Lang , and A. Buchner . 2007. “G*Power 3: A Flexible Statistical Power Analysis Program for the Social, Behavioral, and Biomedical Sciences.” Behavior Research Methods 39, no. 2: 175–191. 10.3758/BF03193146. 17695343

[ejn70239-bib-0021] Friese, U. , M. Köster , U. Hassler , U. Martens , N. Trujillo‐Barreto , and T. Gruber . 2013. “Successful Memory Encoding is Associated With Increased Cross‐Frequency Coupling Between Frontal Theta and Posterior Gamma Oscillations in Human Scalp‐Recorded EEG.” NeuroImage 66: 642–647. 23142278 10.1016/j.neuroimage.2012.11.002

[ejn70239-bib-0022] Godden, D. R. , and A. D. Baddeley . 1975. “Context‐Dependent Memory in Two Natural Environments: On Land and Underwater.” British Journal of Psychology 66, no. 3: 325–331.

[ejn70239-bib-0023] Good, M. A. , P. Barnes , V. Staal , A. McGregor , and R. C. Honey . 2007. “Context‐But Not Familiarity‐Dependent Forms of Object Recognition are Impaired Following Excitotoxic Hippocampal Lesions in Rats.” Behavioral Neuroscience 121, no. 1: 218–223. 17324066 10.1037/0735-7044.121.1.218

[ejn70239-bib-0024] Graetz, S. , J. Daume , U. Friese , and T. Gruber . 2019. “Alterations in Oscillatory Cortical Activity Indicate Changes in Mnemonic Processing During Continuous Item Recognition.” Experimental Brain Research 237, no. 2: 573–583. 10.1007/s00221-018-5439-4. 30488235

[ejn70239-bib-0025] Gruber, T. , and M. M. Müller . 2002. “Effects of Picture Repetition on Induced Gamma Band Responses, Evoked Potentials, and Phase Synchrony in the Human EEG.” Cognitive Brain Research 13, no. 3: 377–392. 10.1016/S0926-6410(01)00130-6. 11919002

[ejn70239-bib-0026] Gruber, T. , D. Tsivilis , C.‐M. Giabbiconi , and M. M. Müller . 2008. “Induced Electroencephalogram Oscillations During Source Memory: Familiarity is Reflected in the Gamma Band, Recollection in the Theta Band.” Journal of Cognitive Neuroscience 20, no. 6: 1043–1053. 18211247 10.1162/jocn.2008.20068

[ejn70239-bib-0027] Guderian, S. , and E. Düzel . 2005. “Induced Theta Oscillations Mediate Large‐Scale Synchrony With Mediotemporal Areas During Recollection in Humans.” Hippocampus 15, no. 7: 901–912. 10.1162/jocn.2008.20068. 16161060

[ejn70239-bib-0028] Guderian, S. , B. H. Schott , A. Richardson‐Klavehn , and E. Düzel . 2009. “Medial Temporal Theta State Before an Event Predicts Episodic Encoding Success in Humans.” Proceedings of the National Academy of Sciences of the United States of America 106, no. 13: 5365–5370. 19289818 10.1073/pnas.0900289106 PMC2663999

[ejn70239-bib-0029] Hanslmayr, S. , T. Staudigl , A. Aslan , and K.‐H. Bäuml . 2010. “Theta Oscillations Predict the Detrimental Effects of Memory Retrieval.” Cognitive, Affective, & Behavioral Neuroscience 10, no. 3: 329–338. 10.3758/CABN.10.3.329 20805534

[ejn70239-bib-0030] Hanslmayr, S. , T. Staudigl , and M.‐C. Fellner . 2012. “Oscillatory Power Decreases and Long‐Term Memory: The Information via Desynchronization Hypothesis.” Frontiers in Human Neuroscience 6: 74. 22514527 10.3389/fnhum.2012.00074 PMC3322486

[ejn70239-bib-0031] Harman, J. , R. Brown , and D. Johnson . 2017. “Improved Memory Elicitation in Virtual Reality: New Experimental Results and Insights.” In *IFIP Conference on Human‐Computer Interaction*, 128–146. Springer International Publishing.

[ejn70239-bib-0032] Hassler, U. , N. Trujillo Barreto , and T. Gruber . 2011. “Induced Gamma Band Responses in Human EEG After the Control of Miniature Saccadic Artifacts.” NeuroImage 57, no. 4: 1411–1421. 10.1016/j.neuroimage.2011.05.062. 21645624

[ejn70239-bib-0033] Hautus, M. J. , N. A. Macmillan , and C. D. Creelman . 2021. Detection Theory: A User's Guide. Routledge.

[ejn70239-bib-0034] Herron, J. E. 2007. “Decomposition of the ERP Late Posterior Negativity: Effects of Retrieval and Response Fluency.” Psychophysiology 44, no. 2: 233–244. 17343707 10.1111/j.1469-8986.2006.00489.x

[ejn70239-bib-0035] Hsieh, L.‐T. , and C. Ranganath . 2014. “Frontal Midline Theta Oscillations During Working Memory Maintenance and Episodic Encoding and Retrieval.” NeuroImage 85: 721–729. 10.1016/j.neuroimage.2013.08.003. 23933041 PMC3859771

[ejn70239-bib-0036] Jacoby, L. L. 1991. “A Process Dissociation Framework: Separating Automatic From Intentional Uses of Memory.” Journal of Memory and Language 30, no. 5: 513–541.

[ejn70239-bib-0037] Johansson, M. , and A. Mecklinger . 2003. “The Late Posterior Negativity in ERP Studies of Episodic Memory: Action Monitoring and Retrieval of Attribute Conjunctions.” Biological Psychology 64, no. 1–2: 91–117. 14602357 10.1016/s0301-0511(03)00104-2

[ejn70239-bib-0038] Johnson, M. K. , S. Hashtroudi , and D. S. Lindsay . 1993. “Source Monitoring.” Psychological Bulletin 114, no. 1: 3–28. 8346328 10.1037/0033-2909.114.1.3

[ejn70239-bib-0039] Junghöfer, M. , T. Elbert , D. M. Tucker , and B. Rockstroh . 2000. “Statistical Control of Artifacts in Dense Array EEG/MEG Studies.” Psychophysiology 37, no. 4: 523–532. 10934911

[ejn70239-bib-0040] Kelter, R. 2020. “Bayesian Alternatives to Null Hypothesis Significance Testing in Biomedical Research: A Non‐Technical Introduction to Bayesian Inference with JASP.” BMC Medical Research Methodology 20, no. 1: 142. 32503439 10.1186/s12874-020-00980-6 PMC7275319

[ejn70239-bib-0041] Khader, P. H. , and F. Rösler . 2011. “EEG Power Changes Reflect Distinct Mechanisms During Long‐Term Memory Retrieval.” Psychophysiology 48, no. 3: 362–369. 20624249 10.1111/j.1469-8986.2010.01063.x

[ejn70239-bib-0042] Kisker, J. , T. Gruber , and B. Schöne . 2021. “Virtual Reality Experiences Promote Autobiographical Retrieval Mechanisms: Electrophysiological Correlates of Laboratory and Virtual Experiences.” Psychological Research 85, no. 7: 2485–2501. 10.1007/s00426-020-01417-x. 32930880 PMC8440245

[ejn70239-bib-0043] Kisker, J. , T. Gruber , and B. Schöne . 2021a. “Experiences in Virtual Reality Entail Different Processes of Retrieval as Opposed to Conventional Laboratory Settings: A Study on Human Memory.” Current Psychology 40: 3190–3197.

[ejn70239-bib-0044] Kisker, J. , M. Johnsdorf , M. Sagehorn , T. Hofmann , T. Gruber , and B. Schöne . 2025. “Visual Information Processing of 2D, Virtual 3D and Real‐World Objects Marked by Theta Band Responses: Visuospatial Processing and Cognitive Load as A Function of Modality.” European Journal of Neuroscience 61, no. 1: e16634. 39648815 10.1111/ejn.16634 PMC11664642

[ejn70239-bib-0045] Kisker, J. , M. Johnsdorf , M. Sagehorn , B. Schöne , and T. Gruber . 2024. “Induced Oscillatory Brain Responses Under Virtual Reality Conditions in the Context of Repetition Priming.” Experimental Brain Research 242: 525–541. 10.1007/s00221-023-06766-8. 38200371 PMC10894769

[ejn70239-bib-0046] Klimesch, W. 1999. “EEG Alpha and Theta Oscillations Reflect Cognitive and Memory Performance: A Review and Analysis.” Brain Research Reviews 29, no. 2–3: 169–195. 10.1016/S0165-0173(98)00056-3. 10209231

[ejn70239-bib-0047] Klimesch, W. , M. Doppelmayr , T. Pachinger , and B. Ripper . 1997. “Brain Oscillations and Human Memory: EEG Correlates in the Upper Alpha and Theta Band.” Neuroscience Letters 238, no. 1–2: 9–12. 10.1016/S0304-3940(97)00771-4. 9464642

[ejn70239-bib-0048] Klimesch, W. , M. Doppelmayr , H. Schimke , and B. Ripper . 1997. “Theta Synchronization and Alpha Desynchronization in a Memory Task.” Psychophysiology 34: 169–176. 10.1111/j.1469-8986.1997.tb02128.x. 9090266

[ejn70239-bib-0049] Köster, M. , and T. Gruber . 2022. “Rhythms of Human Attention and Memory: An Embedded Process Perspective.” Frontiers in Human Neuroscience 16, no. October: 1–20. 10.3389/fnhum.2022.905837. PMC9579292 36277046

[ejn70239-bib-0050] Krokos, E. , C. Plaisant , and A. Varshney . 2019. “Virtual Memory Palaces: Immersion Aids Recall.” Virtual Reality 23, no. 1: 1–15. 10.1007/s10055-018-0346-3.

[ejn70239-bib-0051] Kwon, S. , M. D. Rugg , R. Wiegand , T. Curran , and A. M. Morcom . 2023. “A Meta‐Analysis of Event‐Related Potential Correlates of Recognition Memory.” Psychonomic Bulletin and Review 30, no. 6: 2083–2105). Springer. 10.3758/s13423-023-02309-y. 37434046 PMC10728276

[ejn70239-bib-0052] Lisman, J. E. , and O. Jensen . 2013. “The Theta‐Gamma Neural Code.” Neuron 77, no. 6: 1002–1016. 23522038 10.1016/j.neuron.2013.03.007 PMC3648857

[ejn70239-bib-0053] Lundqvist, M. , E. K. Miller , J. Nordmark , J. Liljefors , and P. Herman . 2024. “Beta: Bursts of Cognition.” Trends in Cognitive Sciences 28, no. 7: 662–676. 10.1016/j.tics.2024.03.010. 38658218

[ejn70239-bib-0054] Lundqvist, M. , J. Rose , P. Herman , S. L. Brincat , T. J. Buschman , and E. K. Miller . 2016. “Gamma and Beta Bursts Underlie Working Memory.” Neuron 90, no. 1: 152–164. 10.1016/j.neuron.2016.02.028. 26996084 PMC5220584

[ejn70239-bib-0055] Macmillan, N. A. 2002. “Signal Detection Theory.” In Stevens' Handbook of Experimental Psychology: Methodology in Experimental Psychology, edited by H. Pashler and J. Wixted , 3rd ed., 49–90. John Wiley & Sons, Inc. 10.1002/0471214426.pas0402.

[ejn70239-bib-0056] Martin, C. B. , D. A. McLean , E. B. O'Neil , and S. Köhler . 2013. “Distinct Familiarity‐Based Response Patterns for Faces and Buildings in Perirhinal and Parahippocampal Cortex.” Journal of Neuroscience 33, no. 26: 10915–10923. 23804111 10.1523/JNEUROSCI.0126-13.2013 PMC6618503

[ejn70239-bib-0057] Mecklinger, A. 2000. “Interfacing Mind and Brain: A Neurocognitive Model of Recognition Memory.” Psychophysiology 37, no. 5: 565–582. 11037034

[ejn70239-bib-0058] Mecklinger, A. , M. Johansson , M. Parra , and S. Hanslmayr . 2007. “Source‐Retrieval Requirements Influence Late ERP and EEG Memory Effects.” Brain Research 1172: 110–123. 17822684 10.1016/j.brainres.2007.07.070

[ejn70239-bib-0059] Mecklinger, A. , T. Rosburg , and M. Johansson . 2016. “Reconstructing the Past: The Late Posterior Negativity (LPN) in Episodic Memory Studies.” Neuroscience & Biobehavioral Reviews 68: 621–638. 27365154 10.1016/j.neubiorev.2016.06.024

[ejn70239-bib-0060] Migo, E. M. , A. R. Mayes , and D. Montaldi . 2012. “Measuring Recollection and Familiarity: Improving the Remember/Know Procedure.” Consciousness and Cognition 21, no. 3: 1435–1455. 22846231 10.1016/j.concog.2012.04.014

[ejn70239-bib-0061] Nyhus, E. , and T. Curran . 2010. “Functional Role of Gamma and Theta Oscillations in Episodic Memory.” Neuroscience and Biobehavioral Reviews 34, no. 7: 1023–1035. 10.1016/j.neubiorev.2009.12.014. 20060015 PMC2856712

[ejn70239-bib-0062] Park, J. L. , and D. I. Donaldson . 2019. “Detecting the Neural Correlates of Episodic Memory With Mobile EEG: Recollecting Objects in the Real World.” NeuroImage 193: 1–9. 30862534 10.1016/j.neuroimage.2019.03.013

[ejn70239-bib-0063] Parks, C. M. 2007. “The Role of Noncriterial Recollection in Estimating Recollection and Familiarity.” Journal of Memory and Language 57, no. 1: 81–100. 18591986 10.1016/j.jml.2007.03.003 PMC2083555

[ejn70239-bib-0064] Pastor, A. , and P. Bourdin‐Kreitz . 2024. “Comparing Episodic Memory Outcomes From Walking Augmented Reality and Stationary Virtual Reality Encoding Experiences.” Scientific Reports 14, no. 1: 7580. 38555291 10.1038/s41598-024-57668-w PMC10981735

[ejn70239-bib-0065] Pastötter, B. , and K.‐H. T. Bäuml . 2014. “Distinct Slow and Fast Cortical Theta Dynamics in Episodic Memory Retrieval.” NeuroImage 94: 155–161. 24632089 10.1016/j.neuroimage.2014.03.002

[ejn70239-bib-0066] Peeters, D. 2018. “A Standardized Set of 3‐D Objects for Virtual Reality Research and Applications.” Behavior Research Methods 50, no. 3: 1047–1054. 10.3758/s13428-017-0925-3. 28646401 PMC5990572

[ejn70239-bib-0067] Pion‐Tonachini, L. , K. Kreutz‐Delgado , and S. Makeig . 2019. “ICLabel: An Automated Electroencephalographic Independent Component Classifier, Dataset, and Website.” NeuroImage 198: 181–197. 10.1016/j.neuroimage.2019.05.026. 31103785 PMC6592775

[ejn70239-bib-0068] Roberts, B. M. , A. Clarke , R. J. Addante , and C. Ranganath . 2018. “Entrainment Enhances Theta Oscillations and Improves Episodic Memory.” Cognitive Neuroscience 9, no. 3–4: 181–193. 30198823 10.1080/17588928.2018.1521386

[ejn70239-bib-0069] Rubo, M. , N. Messerli , and S. Munsch . 2021. “The Human Source Memory System Struggles to Distinguish Virtual Reality and Reality.” Computers in Human Behavior Reports 4: 100111. 10.1016/j.chbr.2021.100111.

[ejn70239-bib-0070] Rugg, M. D. , and T. Curran . 2007. “Event‐Related Potentials and Recognition Memory.” Trends in Cognitive Sciences 11, no. 6: 251–257. 17481940 10.1016/j.tics.2007.04.004

[ejn70239-bib-0071] Sauseng, P. , W. Klimesch , K. F. Heise , et al. 2009. “Brain Oscillatory Substrates of Visual Short‐Term Memory Capacity.” Current Biology 19, no. 21: 1846–1852. 10.1016/j.cub.2009.08.062. 19913428

[ejn70239-bib-0072] Schacter, D. L. 1990 a. “Perceptual Representation Systems and Implicit Memory: Toward a Resolution of the Multiple Memory Systems Debate A.” Annals of the New York Academy of Sciences 608, no. 1: 543–571. 2075961 10.1111/j.1749-6632.1990.tb48909.x

[ejn70239-bib-0073] Schöne, B. , J. Kisker , R. S. Sylvester , E. L. Radtke , and T. Gruber . 2021. “Library for Universal Virtual Reality Experiments (LuVRe): A Standardized Immersive 3D/360° Picture and Video Database for VR Based Research.” Current Psychology 42: 1–5384. 10.1007/s12144-021-01841-1.33519148

[ejn70239-bib-0273] Schöne, B. , M. Wessels , & T. Gruber , (2019). “Experiences in virtual reality: A window to autobiographical memory.” Current Psychology: A Journal for Diverse Perspectives on Diverse Psychological Issues, 38 (3), 715–719. 10.1007/s12144-017-9648-y.

[ejn70239-bib-0074] Serino, S. , and C. Repetto . 2018. “New Trends in Episodic Memory Assessment: Immersive 360° Ecological Videos.” Frontiers in Psychology 9, no. OCT: 1–6. 10.3389/fpsyg.2018.01878. 30333780 PMC6176050

[ejn70239-bib-0075] Shin, Y. S. , R. Masís‐Obando , N. Keshavarzian , R. Dáve , and K. A. Norman . 2021. “Context‐Dependent Memory Effects in Two Immersive Virtual Reality Environments: On Mars and Underwater.” Psychonomic Bulletin & Review 28, no. 2: 574–582. 33201491 10.3758/s13423-020-01835-3 PMC8062363

[ejn70239-bib-0076] Siegel, M. , M. R. Warden , and E. K. Miller . 2009. “Phase‐Dependent Neuronal Coding of Objects in Short‐Term Memory.” Proceedings of the National Academy of Sciences 106, no. 50: 21341–21346. 10.1073/pnas.0908193106. PMC2779828 19926847

[ejn70239-bib-0077] Slater, M. , and S. Wilbur . 1997. “A Framework for Immersive Virtual Environments (FIVE): Speculations on the Role of Presence in Virtual Environments.” Presence Teleoperators and Virtual Environments 6, no. 6: 603–616. 10.1162/pres.1997.6.6.603.

[ejn70239-bib-0078] Smith, S. A. 2019. “Virtual Reality in Episodic Memory Research: A Review.” Psychonomic Bulletin & Review 26: 1213–1237. 10.3758/s13423-019-01605-w. 31037605

[ejn70239-bib-0079] Smith, S. A. , and N. W. Mulligan . 2021. “Immersion, Presence, and Episodic Memory in Virtual Reality Environments.” Memory 29, no. 8: 983–1005. 10.1080/09658211.2021.1953535. 34294002

[ejn70239-bib-0080] Stanley, D. A. , J. E. Roy , M. C. Aoi , N. J. Kopell , and E. K. Miller . 2018. “Low‐Beta Oscillations Turn Up the Gain During Category Judgments.” Cerebral Cortex 28, no. 1: 116–130. 10.1093/cercor/bhw356. 29253255 PMC6248822

[ejn70239-bib-0081] Swets, J. A. , W. P. Tanner Jr. , and T. G. Birdsall . 1961. “Decision Processes in Perception.” Psychological Review 68, no. 5: 301–340. 13774292

[ejn70239-bib-0082] Tallon‐Baudry, C. , and O. Bertrand . 1999. “Oscillatory Gamma Activity in Humans and Its Role in Object Representation.” Trends in Cognitive Sciences 3, no. 4: 151–162. 10322469 10.1016/s1364-6613(99)01299-1

[ejn70239-bib-0083] Tort, A. B. L. , R. Komorowski , H. Eichenbaum , and N. Kopell . 2010. “Measuring Phase‐Amplitude Coupling Between Neuronal Oscillations of Different Frequencies.” Journal of Neurophysiology 104, no. 2: 1195–1210. 20463205 10.1152/jn.00106.2010 PMC2941206

[ejn70239-bib-0084] Tromp, J. , F. Klotzsche , S. Krohn , et al. 2020. “OpenVirtualObjects: An Open Set of Standardized and Validated 3D Household Objects for Virtual Reality‐Based Research, Assessment, and Therapy.” Frontiers in Virtual Reality 1, no. December: 1–8. 10.3389/frvir.2020.611091.

[ejn70239-bib-0085] Tulving, E. 1985. “Memory and consciousness.” Canadian Psychology/Psychologie Canadienne 26, no. 1: 1.

[ejn70239-bib-0086] Tulving, E. , and D. M. Thomson . 1973. “Encoding specificity and retrieval processes in episodic memory.” Psychological Review 80, no. 5: 352.

[ejn70239-bib-0087] Ventura, S. , E. Brivio , G. Riva , and R. M. Baños . 2019. “Immersive Versus Non‐immersive Experience: Exploring the Feasibility of Memory Assessment Through 360 Technology.” Frontiers in Psychology 10, 2509. 31798492 10.3389/fpsyg.2019.02509 PMC6868024

[ejn70239-bib-0088] Wynn, S. C. , C. D. Townsend , and E. Nyhus . 2024. “The Role of Theta and Gamma Oscillations in Item Memory, Source Memory, and Memory Confidence.” Psychophysiology 61: e14602. 38715221 10.1111/psyp.14602 PMC11330366

[ejn70239-bib-0089] Yonelinas, A. P. 2002. “The Nature of Recollection and Familiarity: A Review of 30 Years of Research.” Journal of Memory and Language 46, no. 3: 441–517. 10.1006/jmla.2002.2864.

[ejn70239-bib-0090] Yonelinas, A. P. , and L. L. Jacoby . 1996. “Noncriterial Recollection: Familiarity as Automatic, Irrelevant Recollection.” Consciousness and Cognition 5, no. 1–2: 131–141. 10.1006/ccog.1996.0008 8733927

