# Supplementary material for: Engrams Formed in Virtual Reality Exhibit Reduced Familiarity Upon Retrieval: Electrophysiological Correlates of Source Memory Retrieval Indicate Modality‐Dependent Differences in Recognition Memory
Source: Eur J Neurosci. 2025 Sep 7;62(5):e70239. doi: 10.1111/ejn.70239 (PMC12414874; doi:10.1111/ejn.70239)
Supplement: Supplementary file 1 — Figure S1: Topographical amplitude distribution of the contrasts between engrams with correct source retrieval and correctly rejected items based on the old/new analyses of the FN400 and LPC. Figure S2: Visualization of the induced beta band response. [file EJN-62-0-s001.pdf]

Supplementary Material

Engrams Formed in Virtual Reality Exhibit Reduced Familiarity Upon Retrieval: Electrophysiological Correlates of Source Memory Retrieval Indicate Modality-Dependent Differences in Recognition Memory

S1. *Analyses of the LPC and FN400 for stimuli retrieved with their correct source*

As a countercheck whether our analyses would rather account for the broad central negativity (BCN) than the late posterior negativity (LPN) despite the posterior amplitude distribution, we examined whether items retrieved with a correct source would elicit the canonical frontal and parietal old/new effects, i.e., FN400 and LPC effects. In terms of context familiarity, correctly recognized items with correct source retrieval might result from context familiarity (Addante et al., 2024). Yet it was proposed that correlates of context familiarity do not exhibit both the LPN and FN400 effects, i.e., the correlates of (item) familiarity and recollection.

Consequently, we applied our analyses of the FN400 and LPC to PC-engrams with correct source, VR-engrams with correct source and correctly rejected items. The time windows and electrodes corresponded to those chosen for the comparison of PC-Old, VR-Old and correct rejections, i.e., the analyses of the old/new task. The respective statistics and topographical amplitude distributions can be found below.

Overall, engrams retrieved with correct source exhibited the LPC old/new effect but the FN400 did not reach significance. However, the bayes factor for the latter rmANOVA indicated only anecdotal evidence for the H0, indicating rather absence of evidence for the FN400 rather than evidence of absence.

1 x 3 rmANOVA including the within factor condition (PC-FS, VR-FS, CR)

FN400: $F(2, 56) = 2.61$, $p = .083$, $BF_{10} = 0.763$

LPC: $F(2, 56) = 4.75$, $p = .013$, $\eta^2 = .34$

Post-hoc t-tests comparing the LPC between engrams retrieved with correct source cs. CR

VR-CS vs. CR: $t(28) = 3.74$, $p < .001$, $d = .69$

PC-CS vs. CR: $t(28) = 2.08$, $p = .024$, $d = .39$

Supplementary Figure 1. Topographical amplitude distribution of the contrasts between engrams with correct source retrieval and correctly rejected items based on the old/new analyses of the FN400 and LPC.

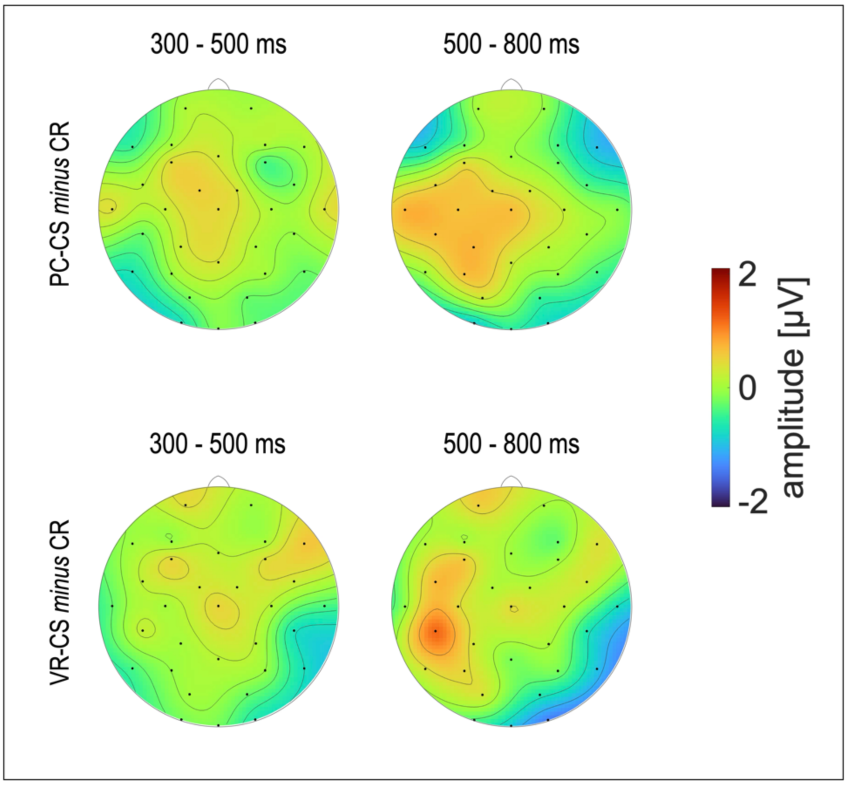

S2. *Analysis of the beta band response*

As suggested by one reviewer, we complemented the spectral power analyses by analyses of the beta band response. A relatively broad range is denoted as beta band (approx. 13 – 35hz), while the specific range of interest can vary across studies (Hanslmayr et al., 2012). Consequently, we plotted the difference between the absolute values of the frequency

response to both kinds of old stimuli (i.e., VR-engrams and PC-engrams) and correctly rejected stimuli (CR) - i.e., abs(mean(VR-Old, PC-Old)) minus abs(CR) - to identify the specific range of interest. The time-frequency data were plotted at electrode Fz and electrode Pz (see Hanslmayr et al. (2012) for a similar procedure), as well as averaged across all electrodes. Based on the time-frequency-plots, a relevant difference in beta power was most pronounced for the 13 – 17 hz range at Pz from 400 – 900 ms after stimulus onset and consequently chosen for analysis (see supplementary figure 2). A previous study comparing the beta band response between correctly recognized stimuli and correctly rejected stimuli indicated the most pronounced effects at positions at left temporal-parietal electrodes, with a broader distribution for objects (Hanslmayr et al., 2012; Khader & Rösler, 2011). Consequently, Pz, PO3, CP1, P3, C3 and CP5 were chosen for analyses.

A 1 x 3 rmANOVA including the within-factor (Item-)*Condition* (PC-Old, VR-Old, CR) was carried out with respect to the old/new task and a 1 x 5 rmANOVA including the factor (Source-)*Condition* (PC-CS, PC-FS, VR-CS, VR-FS, CR) was carried out regarding the source identification task. Since these analyses were not planned a priori but were added during the review process, all tests were carried out two-tailed and FDR-corrected (Benjamini & Hochberg, 1995). However, based on the literature (Hanslmayr et al., 2012; Khader & Rösler, 2011), it could be expected that old stimuli would be associated with a more negative beta response compared to correctly rejected stimuli.

With respect to the old/new task, a significant effect of (Item-)*Condition* was found $F(1.50, 44.86) = 5.73$, $p = .011$, $\eta 2 = .16$). Post-hoc *t*-tests revealed old/new effects, i.e., a more negative beta response to old stimuli than to CR regarding both kinds of engrams (VR-Old vs. CR: $t(30) = -2.45$, $p = .020$, d = .44; PC-Old vs. CR: $t(30) = -2.66$, $p = .013$, d = .48). Yet no significant difference was found between VR- and PC-engrams ($t(30) = -0.94$, $p =$ .353).

Regarding the source identification task, no significant effect of (Source-)*Condition* on the beta band response was found ($F(4,100) = 2.97$, $p = .097$, $BF_{10} = 0.34$).

Supplementary Figure 2. Visualization of the induced beta band response.

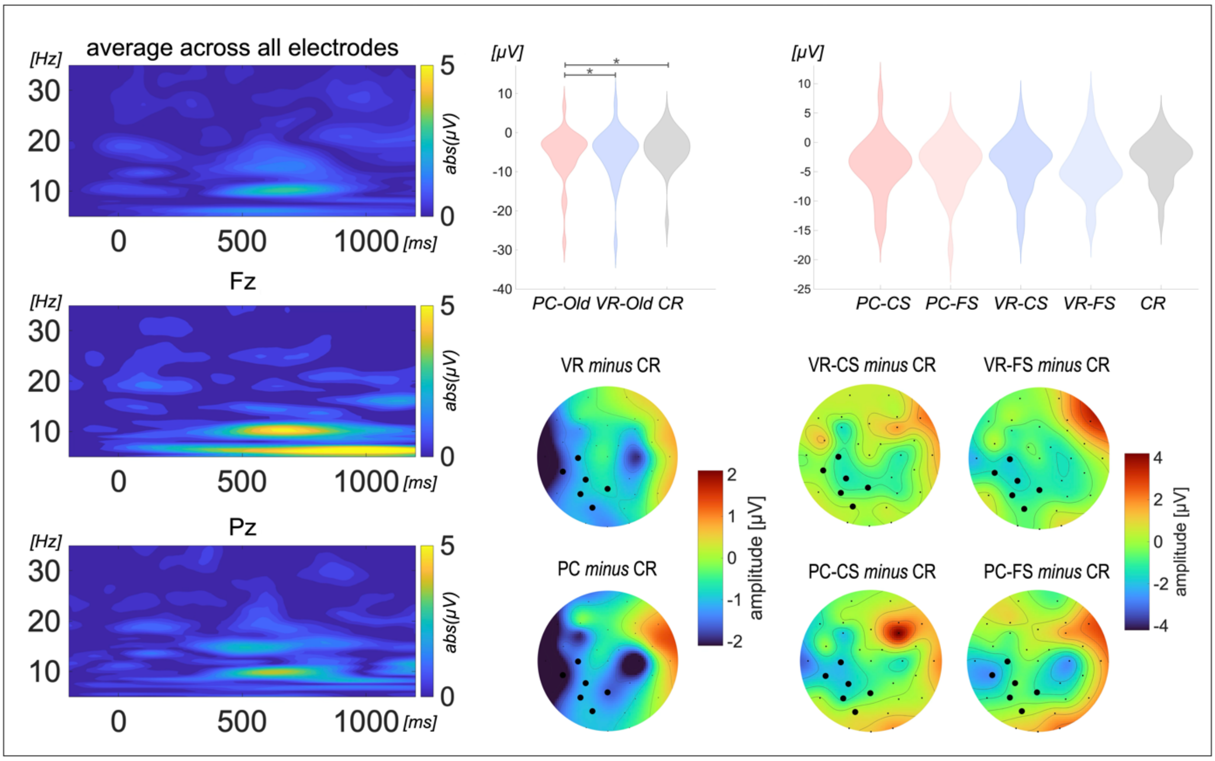

Note. The time-frequency plots depict the difference between the absolute frequency response to old stimuli (i.e., mean of VR-engrams and PC-engrams) minus the absolute frequency response to correct rejections separately for Fz and Pz, as well as averaged across all electrodes. The electrodes used for statistical comparisons were chosen based on previous literature (Hanslmayr et al., 2012; Khader & Rösler, 2011) and are marked in the respective mean topographies as black dots (Pz, PO3, CP1, P3, C3 and CP5). The violin plots indicate each conditions distribution. VR-CS = VR-encoded item with correct source identification, VR-FS = VR-encoded item with false source identification, PC-CS = PC-encoded item with correct source identification, PC-FS = PC-encoded item with false source identification, CR = Correct rejection. Comparisons significant after *fdr*-correction are indicated as follows: *$p < .02$

References*

Addante, R. J., Clise, E., Waechter, R., Bengson, J., Drane, D. L., & Perez-Caban, J. (2024). Context familiarity is a third kind of episodic memory distinct from item familiarity and recollection. *Iscience*, *27*(12).

Benjamini, Y., & Hochberg, Y. (1995). Controlling the false discovery rate: a practical and powerful approach to multiple testing. *Journal of the Royal Statistical Society: Series B (Methodological)*, *57*(1), 289–300.

Hanslmayr, S., Staudigl, T., & Fellner, M.-C. (2012). Oscillatory power decreases and long-term memory: the information via desynchronization hypothesis. *Frontiers in Human Neuroscience*, *6*, 74.

Khader, P. H., & Rösler, F. (2011). EEG power changes reflect distinct mechanisms during long-term memory retrieval. *Psychophysiology*, *48*(3), 362–369.

**these references can also be found in the main text of the manuscript*
